# Multi-omics revealed rumen microbiota metabolism and host immune regulation in Tibetan sheep of different ages

**DOI:** 10.3389/fmicb.2024.1339889

**Published:** 2024-02-13

**Authors:** Yuzhu Sha, Xiu Liu, Yanyu He, Shengguo Zhao, Jiang Hu, Jiqing Wang, Wenhao Li, Pengyang Shao, Fanxiong Wang, Xiaowei Chen, Wenxin Yang, Zhuanhui Xie

**Affiliations:** ^1^College of Animal Science and Technology/Gansu Key Laboratory of Herbivorous Animal Biotechnology, Gansu Agricultural University, Lanzhou, China; ^2^School of Fundamental Sciences, Massey University, Palmerston North, New Zealand; ^3^Academy of Animal Science and Veterinary Medicine, Qinghai University, Xining, China

**Keywords:** Tibetan sheep, rumen microbiota, metabolites, ages, immune

## Abstract

The rumen microbiota and metabolites play an important role in energy metabolism and immune regulation of the host. However, the regulatory mechanism of rumen microbiota and metabolite interactions with host on Tibetan sheep’s plateau adaptability is still unclear. We analyzed the ruminal microbiome and metabolome, host transcriptome and serum metabolome characteristics of Tibetan sheep at different ages. Biomarkers *Butyrivibrio*, *Lachnospiraceae_XPB1014_group*, *Prevotella*, and *Rikenellaceae_RC9_gut_group* were found in 4 months, 1.5 years, 3.5 years, and 6 years Tibetan sheep, respectively. The rumen microbial metabolites were mainly enriched in galactose metabolism, unsaturated fatty acid biosynthesis and fatty acid degradation pathways, and had significant correlation with microbiota. These metabolites further interact with mRNA, and are co-enriched in arginine and proline metabolism, metabolism of xenobiotics by cytochrome P450, propanoate metabolism, starch and sucrose metabolism, gap junction pathway. Meanwhile, serum metabolites also have a similar function, such as chemical carcinogenesis − reactive oxygen species, limonene and pinene degradation, and cutin, suberine and wax biosynthesis, thus participating in the regulation of the body’s immune and energy-related metabolic processes. This study systematically revealed that rumen microbiota, metabolites, mRNA and serum metabolites of Tibetan sheep were involved in the regulation of fermentation metabolic function and immune level of Tibetan sheep at different ages, which provided a new perspective for plateau adaptability research of Tibetan sheep at different ages.

## Introduction

Tibetan sheep, as a unique ruminant and dominant germplasm resource in the Qinghai-Tibet Plateau, has excellent production performance and adaptability in the special plateau environment. Relying on natural grassland resources, Tibetan sheep graze on the alpine pasture all the year around, taking natural pasture as the main source of nutrients, and providing metabolites for survival and production through the powerful ruminal fermentation function, so as to adapt to the extreme and harsh plateau environment (Jing et al., 2020). The rumen is an important marker organ of ruminants and is rich in microbiota such as bacteria, protozoa and fungi, which have coevolved with the host to influence the phenotype and play important metabolic, digestive and immune roles in the host (Tremaroli and Backhed, 2012). Microbiota can decompose cellulose, hemicellulose and other indigestible substances in the host body and produce short-chain fatty acids (SCFAs), microbial proteins, ammonia and other metabolites (Rey et al., 2014), which play an important role in host immune regulation (Martin et al., 2010), disease prevention (Tlaskalova-Hogenova et al., 2011), energy balance (Shabat et al., 2016), and physiological development (Sommer and Backhed, 2013). Thus, microbiota affects the survival, production and reproduction ability of ruminants in specific environments. Among them, 50–85% of SCFAs are directly absorbed through the rumen epithelium as the main energy source of ruminants (Aschenbach et al., 2011), and affect the expression of host genes related to energy metabolism, so as to adapt to the extreme plateau environment through co-evolution with the genome (Zhang et al., 2016). In addition, the rumen of ruminants is not only an important organ of fermentation and metabolism, but also one of the important immune barriers, mainly composed of microbial barriers, physical barriers and immune barriers, which can prevent the transmission of pathogens or toxins to host tissues (Shen et al., 2019). In addition to energy supply, ruminal metabolites also participate in the regulation of various physiological functions of the host, such as the regulation of ruminal epithelial growth, insulin secretion and immune response (Kristensen and Harmon, 2004; Gorka et al., 2009; Kim et al., 2013). SCFAs can also stimulate the activation of the sympathetic nervous system, promote the body’s energy consumption (Kim et al., 2013), and reduce fat synthesis (Bergman, 1990). All these studies show that there is a certain connection between gastrointestinal microbiota and host function, and host genetic structure and microbiota together affect host metabolic phenotype.

Rumen microbiota is crucial for host health and growth performance, and there are certain differences in host gastrointestinal microbial composition and function at different developmental stages. From birth to 2 years old, the structure of rumen microbiota of cows has changed significantly, which leads to changes in rumen ecosystem (Jami et al., 2013), and then affects the performance of cows. Yin’s study on the development of rumen bacteria in lambs from birth to 4 months of age found that the metabolism level, fermentation level, bacterial community and its function were all affected by the age of lambs, and the rumen microbial community and function were unstable before the age of 20 days (Yin et al., 2021). In addition, there are certain differences in microbiota and metabolites between 1-month-old and 6-month-old Tibetan sheep (Li et al., 2020), and it has been found that age-related microbial changes are closely related to host inflammation (Zhang et al., 2019). Changes in intestinal microbiota of old cows led to changes in ruminal fermentation mode, reduced functions related to carbohydrate and lipid metabolism, and increased levels of inflammatory factors compared with young cows. Studies have found that changes in the structure and physiological characteristics of the rumen are related to the development of rumen microbiota with the increase of age, and their fermented metabolites are crucial for the development of rumen wall papillae (Beharka et al., 1998). Our previous study also proved that there are certain differences in ruminal fermentation metabolites SCFAs in Tibetan sheep of different ages, which lead to changes in the structure and gene expression of the rumen epithelium (Sha et al., 2022), thereby affecting the development and function of the rumen epithelium and mediating the adaptability of Tibetan sheep of different ages to the plateau environment. The adaptability of plateau animals to environmental stress is different in different growth and development stages, which leads to differences in their fermentation and metabolism functions. However, it is not clear about the changes of rumen microbiota and metabolites during the whole life stage of Tibetan sheep from lamb to youth, adulthood and old age, and how they affect host gene expression and metabolism through rumen microbial fermentation, and then adapt to the plateau environment. Therefore, this study will compare and analyze the rumen microbiota and their metabolites, host genes and serum metabolites of Tibetan sheep at different ages, clarify the differences in rumen microbial fermentation metabolism and host immune function in the whole life stage of Tibetan sheep from lamb to youth, adulthood and old age, and reveal the response of Tibetan sheep at different ages to the pressure of plateau environment. It provides a reference for the feeding and management of Tibetan sheep at different ages.

## Materials and methods

### Experimental design and sample collection

The grazing Tibetan sheep in Haiyan County, Haibei Prefecture, Qinghai Province, China (Altitude 3,500 m) were selected as the study subjects. Twenty-four Tibetan sheep of different ages (Euler type, ♀) were randomly selected from the flock of the same herder, which were as follows: 4 months (4 M, *n* = 6, representing lambs), 1.5 years (1.5 Y, *n* = 6, representing young sheep), 3.5 years (3.5 Y, *n* = 6, representing adult sheep), and 6 years (6 Y, *n* = 6, representing old sheep). All the experimental sheep were in the local traditional natural grazing management state, without any supplementary feeding, and all the ewes were nonpregnant. The types of pasture grass and nutritional level are shown in Supplementary Table S1. The collection of animal samples is approved by the ethics committee (approval no. GAU-LC-2020-27). Before grazing in the morning, the jugular vein blood of all experimental sheep was collected by vacuum tube, centrifuged (5,000 ×*g*, 20 min, 4°C), and the serum was separated and stored at −20°C for the determination of serum non-targeted metabolomics. Subsequently, slaughter (rapid neck exsanguination) was carried out in accordance with local tradition and in compliance with the requirements of the ethics committee. After the rumen is removed, rumen fluid was collected and packaged into frozen tubes and stored in liquid nitrogen for subsequent 16S rRNA sequencing and non-targeted metabolomics determination. The epithelial tissue samples from the ventral sac of the rumen were cut out, rinsed with PBS, and stored in liquid nitrogen in frozen tubes for RNA-sequencing.

### DNA extraction and 16S rRNA sequencing

A MN NucleoSpin 96 Soil kit (Macherey-Nagel, Germany) was used to extract microbial DNA from the samples of rumen contents, and the concentration and purity were detected by NanoPhotometer (N60, Germany). Through PCR amplification of the V3-V4 region of the 16S rRNA gene, the community structure of the rumen microbiota was obtained. The primers were (338F 5’-ACTCCTACGGGAGGCA GCAG-3′ and 806R 5’-GGACTACHVGGGTWTCTAAT-3′). The library obtained by this PCR amplification process was then sequenced on an Illumina MiSeq platform (Illumina, San Diego, CA, United States), and the bioinformatics analysis was performed using BMKCloud.^1^ The raw data returned by the Illumina MiSeq platform were subjected to merging of paired-end reads (FLASH v1.2.7), filtering (Trimmomatic v0.33), and remove chimera (UCHIME v4.2) in order to obtain optimized sequences (tags). The Usearch software (Edgar, 2013) was used to cluster Tags at 97% similarity level, obtain OTU, and perform taxonomic annotation of OTU based on Silva (bacteria) taxonomic database. Based on the OTU analysis results, the samples were analyzed at each taxonomic level, and the community structure map of each sample at the phylum, class, order, family, genus and species taxonomic level was obtained. Alpha diversity was used to analyze the species diversity within a single sample, and Alpha diversity index Ace, Chao1, Shannon and Simpson index were obtained, and sample dilution curve (Wang et al., 2012) and rank abundance curve were plotted. Beta diversity analysis was used to compare the differences in species diversity (composition and structure of the microbiota) between different samples. The PCA map of the sample was obtained according to the distance matrix (Dubois et al., 2010), and the Biomarker with statistical differences between different groups was found by the significance analysis between groups (LEfSe analysis) (Segata et al., 2011). Metastats software was used to perform *t*-test on species abundance data between groups to obtain *p* values (White et al., 2009), and *q* values were obtained by correcting *p* values. Finally, the species that caused the difference in the composition of the two groups of samples were screened out according to the *q* value. 16S gene function analysis was performed by Kyoto Encyclopedia of Genes and Genomes (KEGG) and Clusters of Orthologous Groups (COG).

### LS-MS/MS metabolic spectrometry determination

Metabolites in rumen contents and serum of 24 Tibetan sheep were detected by liquid chromatograph - mass spectrometry. The samples were thawed at room temperature and pre-processed according to Dunn et al. (2011), Want et al. (2010), Dunn et al. (2011), and Want et al. (2010), and samples were prepared according to the method of Liu et al. (2022). Finally, 10 μL was mixed into QC samples for machine detection. The detection platform was Waters Acquity I-Class PLUS ultra-performance liquid chromatography coupled with Waters Xevo G2-XS QTOF high resolution mass spectrometry. The chromatographic column was Acquity UPLC HSS T3 column (1.8 μm, 2.1*100 mm) purchased from Waters, and the sample detection parameters were according to the method of Liu et al. (2022). The original data collected by MassLynx V4.2 were processed by Progenesis QI software for peak extraction, peak alignment and other data processing operations. The identification was carried out based on the online METLIN database of Progenesis QI software, the public database and the self-built database of BMK company, and the theoretical fragment identification was carried out at the same time. The deviation of the parent ion mass number was within 100 ppm, and the deviation of the fragment ion mass number was within 50 ppm. Bioinformatics analysis was carried out on the identified metabolites through the BMKCloud platform (footnote 1), and the differential metabolites were screened by combining the multiple of difference, *p* value of *t*-test and VIP (Variable Importance in Projection) value of OPLS-DA model. With FC (Fold Change) >2. *p* < 0.01 and VIP > 1 as the screening criteria, differential metabolite analysis was performed, and KEGG functional annotation and enrichment analysis were performed on differential metabolites (Kanehisa and Goto, 2000).

### RNA extraction and RNA-sequencing

TRIzol reagent (Invitrogen, DP662-T1C) was used to extract total RNA from rumen epithelial tissue of Tibetan sheep. Nanodrop2000 (v1, Thermo) was used for con-centration detection. Agient2100, LabChip GX (platinum, the model Platinum Platinum Elmer LabChip GX) was tested for integrity. cDNA libraries were constructed using the VAHTS Universal V6 RNA-seq Library Prep Kit for Illumina® kit (NR604-02). VAHTSTM DNA Clean Beads kit (N411-03) was further used for product purification. The constructed library was sequenced by illumina novaseq6000 (San Diego), and bioinformatics analysis was performed on BMKCloud (footnote 1). After Data filtering, Clean Data was obtained. Sequence alignment between Clean Data and reference genome *Ovis_aries* (Oar_rambouillet_v1.0) was performed by HISAT (Li et al., 2015). The Mapped Data was obtained and the reads on the pairs were assembled using StringTie (Kim et al., 2015). FPKM (Trapnell et al., 2010) (Fragments Per Kilobase of transcript per Million fragments mapped) was used as an indicator to measure the expression level of transcripts or genes. DESeq2 data analysis method was used to analyze differentially expressed genes, Fold Change (FC) represents the ratio of expression levels between two groups, False Discovery Rate (FDR) is obtained by correcting the difference significance value of *p*, with FC ≥ 2 and FDR < 0.01 was used as the screening criterion for differentially expressed genes.

### Data analysis

According to the method of McHardy et al. (2013), the joint analysis of microbiome and metabolome was carried out, and PCoA was used to reduce the dimension of microbiome (genus level) and metabolome. Firstly, the distance matrix was calculated by using the microbial quantitative matrix and the metabolite quantitative matrix respectively, where the distance algorithm for the microbiome was Bayesian distance, and the distance algorithm for the metabolome was Euclidean distance. PCoA was used to rank the distance. The coordinates of the feature axes in the PCoA results of the microbiome and the metabolome were extracted, and Procrustes analysis was performed to compare the similarity and variation between the microbiome and the metabolome. Weighted gene co-expression network analysis (WGCNA) is used to reduce the dimension of metabolic data, and metabolites are divided into different metabolite clusters. The expression levels of metabolite clusters are expressed by the median content in the same cluster. Pearson correlation analysis was performed with microorganisms, heat map was drawn, and the results of correlation analysis were screened. The screening conditions were *p* values, and the standards were: CCP < 0.05, then count the frequency of metabolite clusters/microorganisms, table the correlation results of metabolite clusters/microorganisms with top30 frequency, and draw chord diagram. WGCNA (Zhang et al., 2013) dimension reduction analysis was performed on transcriptome and metabolome data, and genes and metabolites were divided into different modules. The eigengene of the corresponding module represented the content of gene/metabolite module, and the correlation between transcriptome module and metabolome module was calculated after dimension reduction. Retain data containing at least one set of correlations with a *p*-value of CCP<0.05, and then the heat map was drawn. The pathways involved in genes in the transcriptome and metabolites in the metabolome were compared to obtain the number of common participating pathways, and Venn diagram was drawn (Chen and Boutros, 2011). The top 10 KEGG pathways with the largest number of common participating genes and metabolites identified in this experiment were counted for visual analysis.

## Results

### Characteristics of rumen microbiota of Tibetan sheep at different ages

**Figure 1:**
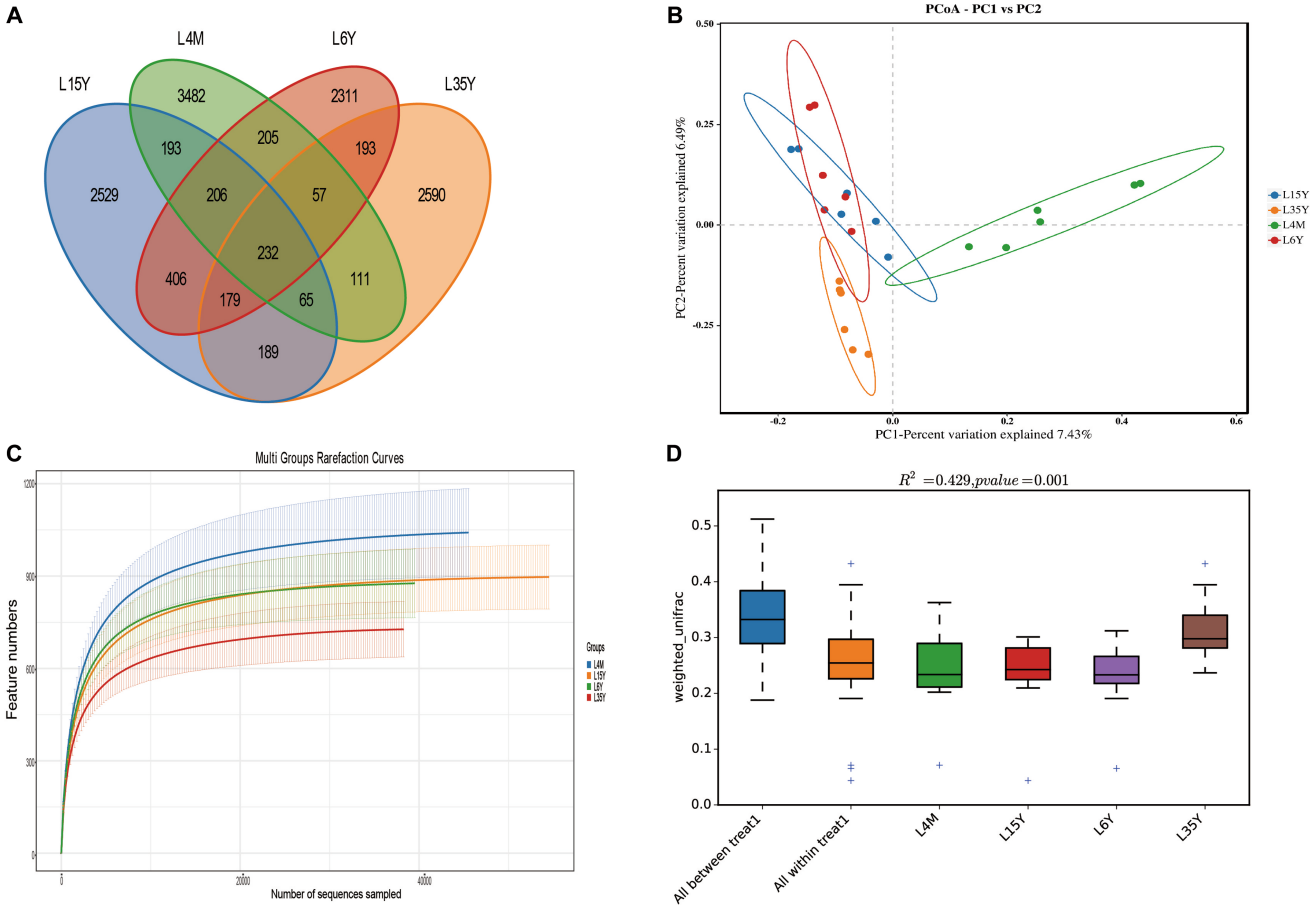
Rumen microbiota diversity of Tibetan sheep at different ages. **(A)** Distribution of OUT; **(B)** PCoA analysis; **(C)** dilution curve; **(D)** Anosim analysis.

A total of 1,800,295 pairs of reads were obtained from rumen microbiota of Tibetan sheep at different ages, and an average of 74,724 clean reads were generated for each sample after quality control and splicing. OTU clustering analysis showed that a total of 3,482 (4 M), 2,529 (1.5 Y), 2,590 (3.5 Y) and 2,311 (6 Y) unique outs were obtained for the four age groups (Figure 1A). The dilution curve flattens out at 40,000 reads, which ensures the validity of sequencing (Figure 1C). PCoA and Anosim analysis show that there are certain differences in ruminal microbiota in the four age groups (Figures 1B,D), and the differences between groups are greater than the differences within groups. Alpha diversity analysis (Table 1) showed that the Shannon index of 4 M was significantly larger than 1.5 Y and 3.5 Y (*p* < 0.05), Simpson index was significantly greater than 3.5 Y (*p* < 0.05), ACE and Chao1 index in 4 M were significantly higher than 1.5 Y and 3.5 Y (*p* < 0.05).

**Table 1:** Rumen microbial diversity index of Tibetan sheep at different ages.

	L4 M	L1.5 Y	L3.5 Y	L6 Y
Shannon	8.622 ± 0.107^a^	8.254 ± 0.131^b^	7.913 ± 0.133^c^	8.366 ± 0.077^ab^
Simpson	0.994 ± 0.001^a^	0.992 ± 0.001^ab^	0.990 ± 0.001^b^	0.993 ± 0.000^ab^
ACE	1052.667 ± 60.102^a^	900.432 ± 43.006^b^	731.539 ± 36.203^c^	885.124 ± 48.272^b^
Chao1	1050.674 ± 59.883^a^	899.216 ± 42.786^b^	730.2784 ± 36.113^c^	884.352 ± 48.252^b^
PD_whole_tree	85.558 ± 3.251^a^	79.976 ± 2.672^ab^	71.716 ± 2.632^b^	78.635 ± 2.201^ab^

Different superscript lowercase letters indicate significant differences on the same line, *p* < 0.05.

**Figure 2:**
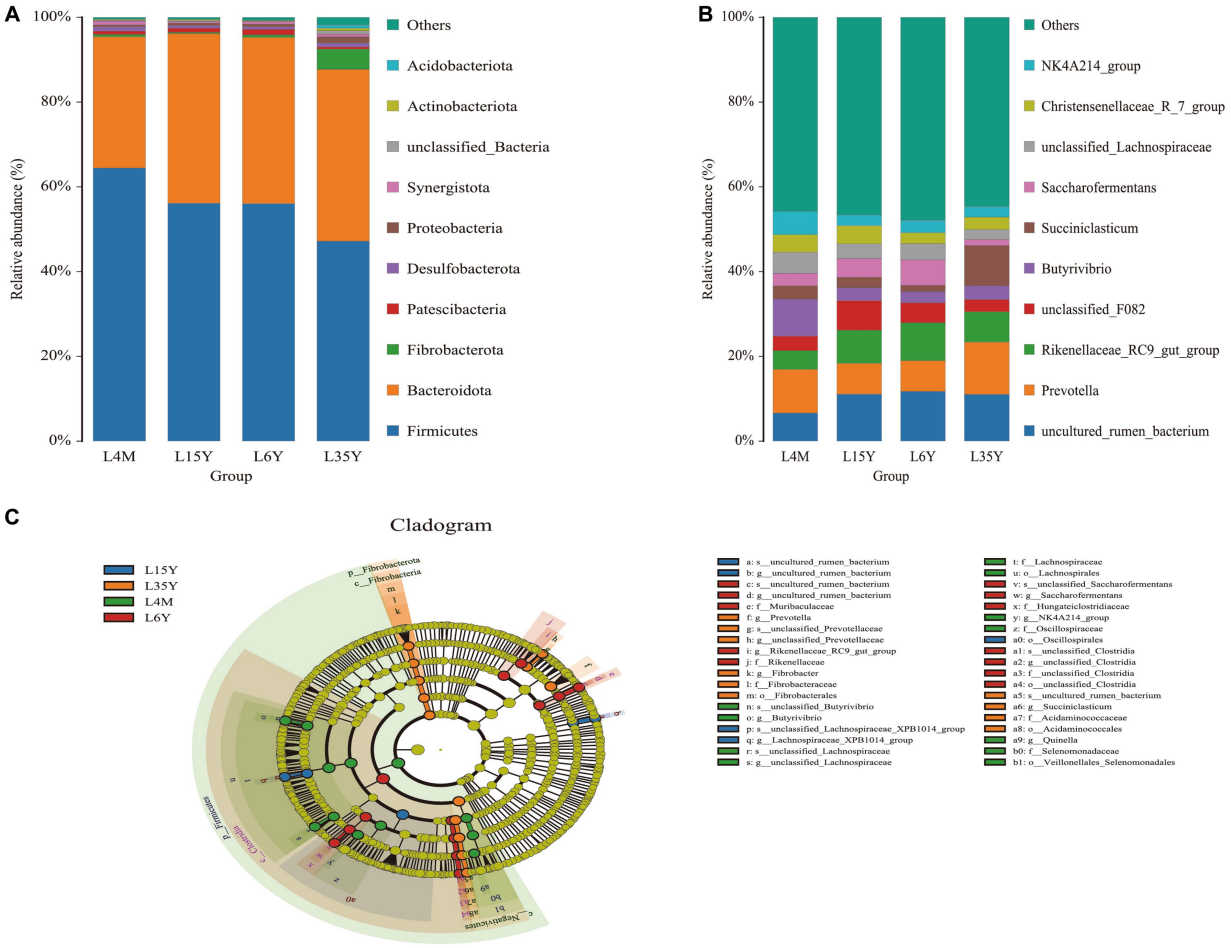
Rumen microbial composition and LEfSe analysis of Tibetan sheep at different ages. **(A)** Species composition at the phylum level; **(B)** Species composition at the genus level; **(C)** LEfSe analysis.

**Figure 3:**
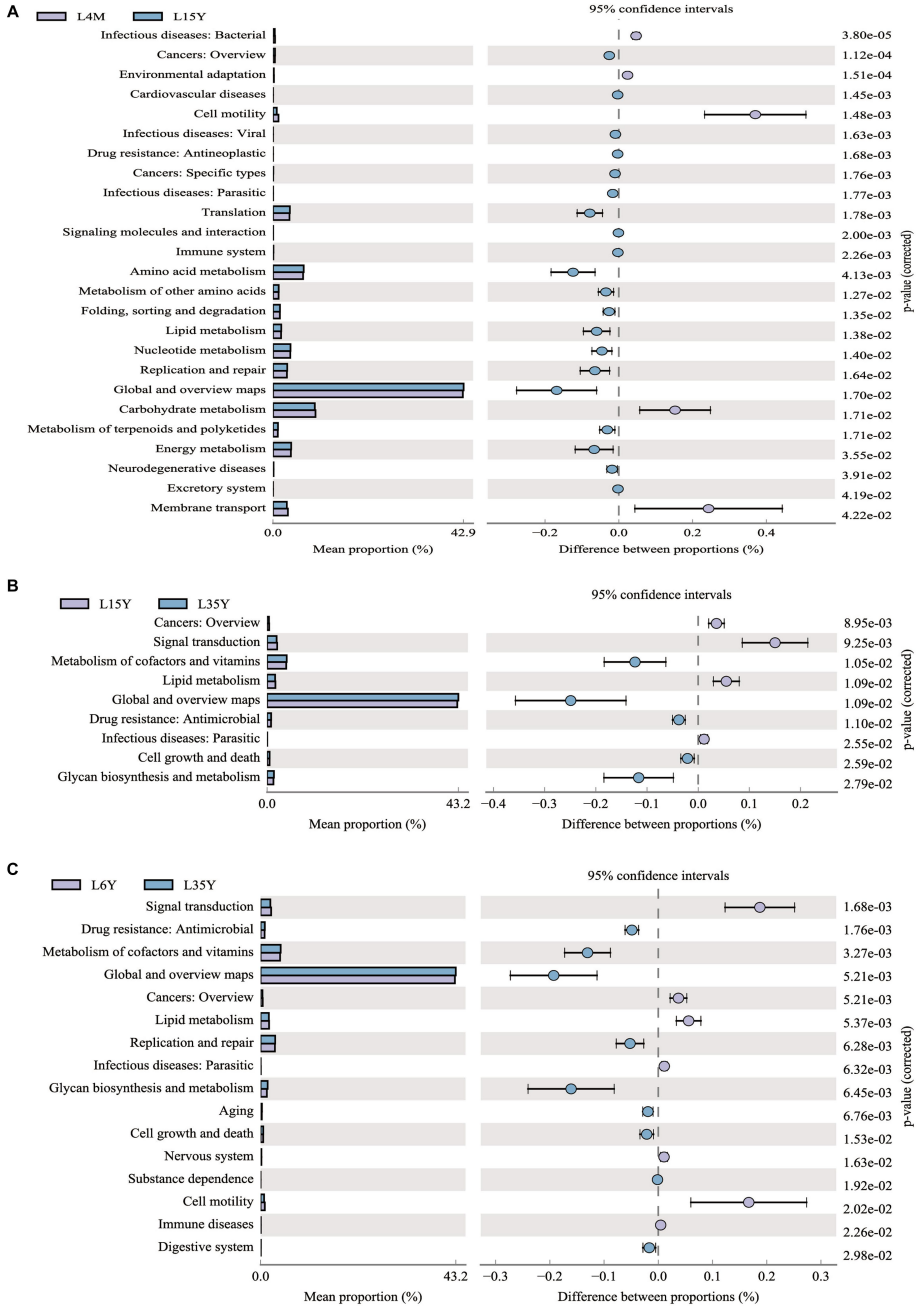
KEGG functional prediction analysis of rumen microbiota in Tibetan sheep at different ages. **(A)** 4 M_vs_1.5 Y; **(B)** 1.5 Y_vs_3.5 Y; **(C)** 3.5 Y_vs_6 Y.

At the phylum level (Figure 2A), *Firmicutes* decreased with age, reached the minimum at 3.5 Y, and increased again at 6 Y. *Bacteroidota* had the highest abundance in 1.5 Y and 3.5 Y, and was significantly lower in 4 M than in 1.5 Y (*p* < 0.05). *Fibrobacterota* was significantly higher in 6 Y than in other age groups (*p* < 0.05). At the genus level (Figure 2B), *Prevotella* was most abundant in 4 M and 3.5 Y, and was significantly higher in 3.5 Y than in 1.5 Y and 6 Y (*p* < 0.05), *Rikenellaceae_RC9_gut_group* had the highest abundance in 6 Y, which was significantly higher than that in 4 M (*p* < 0.05), but not significantly different from 1.5 Y and 3.5 Y (*p* > 0.05). LEfSe analysis found biomarkers of different age groups (Figure 2C), *Butyrivibrio*, *NK4A214_group* and *Quinella* in 4 M, *Lachnospiraceae_XPB1014_group* in 1.5 Y, *Prevotella* and *Succiniclasticum* in 3.5 Y, *Rikenellaceae_RC9_gut_group* and *Saccharofermentans* in 6 Y. KEGG function prediction analysis found (Figure 3) that amino acid metabolism and energy metabolism functions were significantly increased in 4 M_vs_1.5 Y (*p* < 0.05), while carbohydrate metabolism function reached the highest in 4 M. In addition, metabolism of cofactors and vitamins at 3.5 Y was significantly higher than that at 1.5 Y and 6 Y (*p* < 0.05).

### Characteristics of microbiota metabolic profile in rumen of Tibetan sheep at different ages

There were certain differences in rumen microbiota metabolites of Tibetan sheep at different ages (Supplementary Figure S1; Supplementary Data S1). A total of 1,206 differential metabolites were found in 4 M_vs_1.5 Y (Supplementary Figure S2A), and analysis of metabolites with smaller *p* values showed that Decanoic acid was significantly down-regulated at 1.5 years of age (*p* < 0.05), while 3’-DEOXY-3’-FLUOROTHYMIDINE, 2-Succinylbenzoate, Cys Tyr Cys Trp, DG (14:0/16:1(9Z)/0:0) were significantly upregulated (*p* < 0.05). Two hundred and Eighteen differential metabolites were found in 1.5 Y_vs_3.5 Y (Supplementary Figure S2B), among which Bafilomycin A1(Baf-A1) was significantly upregulated at 3.5 Y (*p* < 0.05), while Hovenine A,1, 2-dioctanoyl PC, Aminopentol,1,3,5,7,9,11, 13-pentadecaheptaene were significantly down-regulated (*p* < 0.05). Five hundred and seven differential metabolites were found in 3.5 Y_vs_6 Y (Supplementary Figure S2C), among which Leucopelargonidin 3-O-alpha-L-rhamno-beta-D-glucopyranoside was significantly down-regulated at 6 Y (*p* < 0.05), and Oleanolic acid, 1-Pentadecene, 4-hydroxy-5 -(3′,4′-dihydroxyphenyl)-valeric acid-O-glucuronide and 3-oxo-7,8-dihydro-alpha-ionol were significantly up-regulated (*p* < 0.05). 1,444 differential metabolites were identified in 4 M_vs_6 Y (Supplementary Figure S2D), among which Megalomicin B was significantly down-regulated at 6 Y (*p* < 0.05), 3-oxo-7,8-dihydro-alpha-ionol, 1-Phenyl-2-decanoylamino-3-morpholino-1-propanol, alpha-Valerenol, Tetrahydropersin were significantly up-regulated (*p* < 0.05).

**Figure 4:**
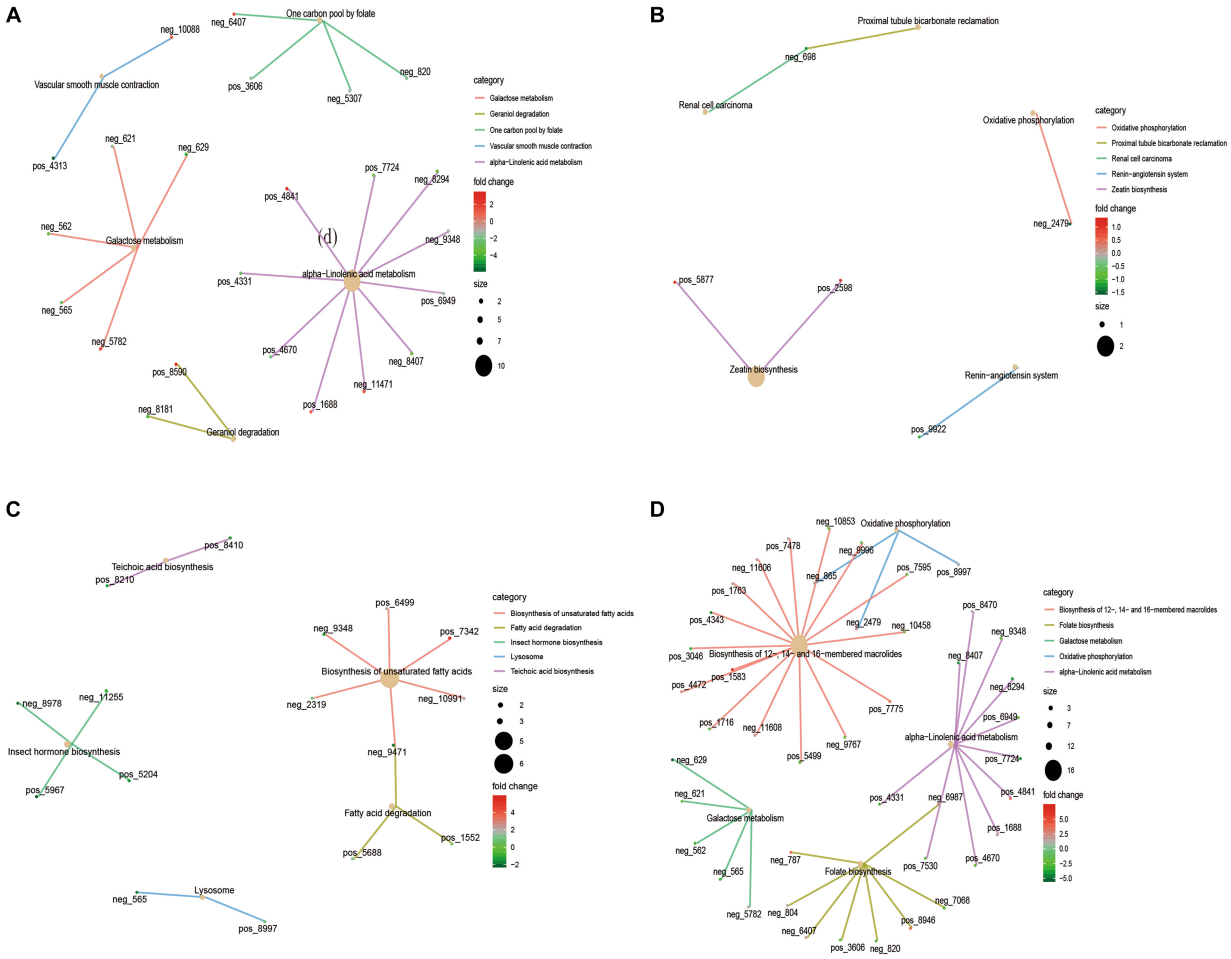
KEGG functional enrichment analysis of rumen microbial metabolites in Tibetan sheep at different ages. **(A)** 4 M_vs_1.5 Y; **(B)** 1.5 Y_vs_3.5 Y; **(C)** 3.5 Y_vs_6 Y; **(D)** 4 M_vs_6 Y.

KEGG functional enrichment analysis of differential metabolites showed that galactose metabolism and alpha−linolenic acid metabolism were mainly enriched in 4 M_vs_1.5 Y (Figure 4A). In Galactose metabolism, D-Mannose (neg-565), myo-Inositol (neg-562) and Raffinose (neg-629) were down-regulated at 1.5 Y, while Melibiitol was up-regulated. In 1.5 Y_vs_3.5 Y (Figure 4B), the enriched functions were less, mainly in oxidative phosphorylation. In the 3.5 Y_vs_6 Y (Figure 4C), it was primarily enriched in the biosynthesis of unsaturated fatty acids and fatty acid degradation. In the 4 M_vs_6 Y (Figure 4D), it was primarily enriched in galactose metabolism, alpha-linolenic acid metabolism, and folate biosynthesis.

### Interaction analysis of rumen microbiota and their metabolites in Tibetan sheep at different ages

**Figure 5:**
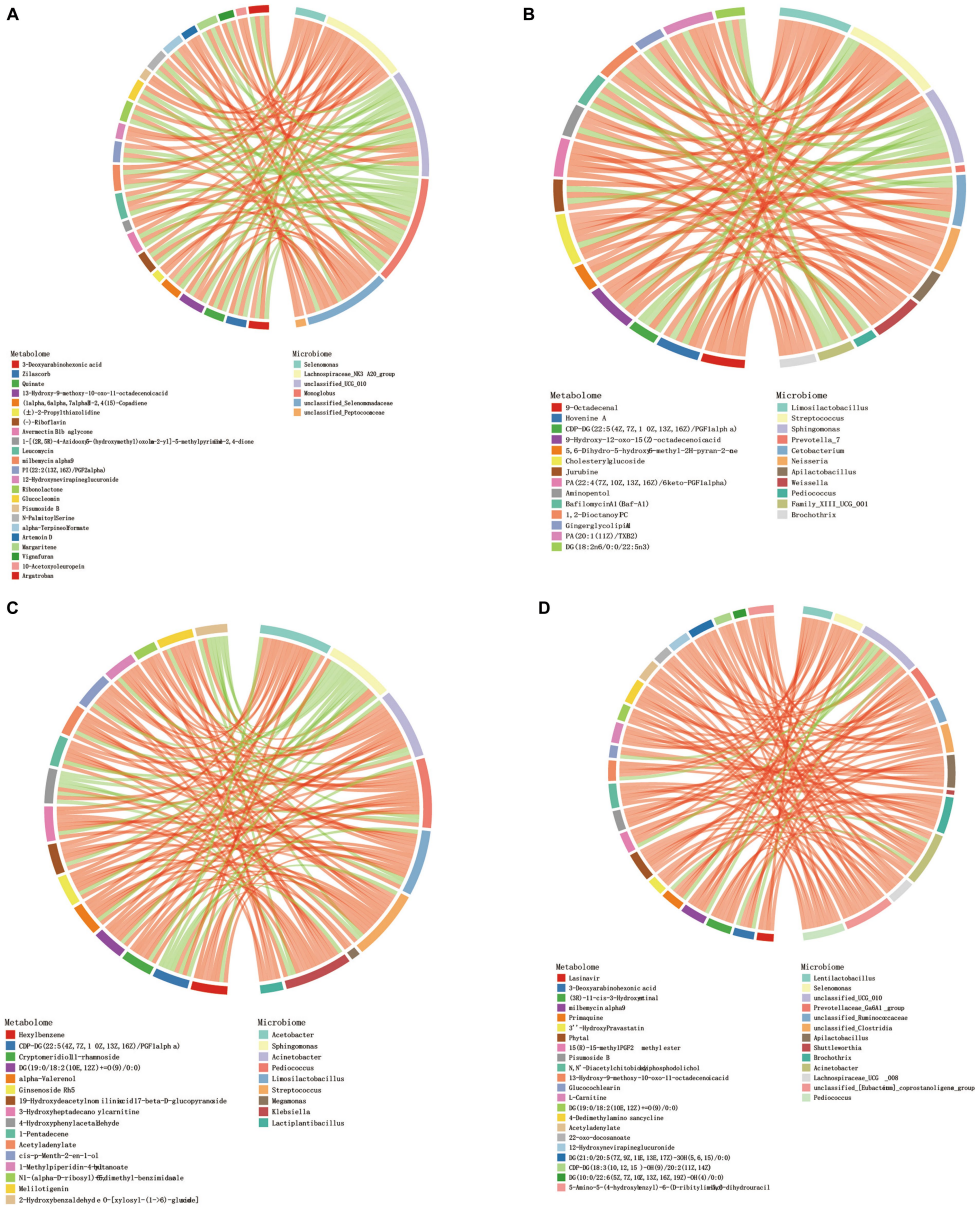
Chord diagram analysis of the correlation between differential metabolites and differential flora. **(A)** 4 M_vs_1.5 Y; **(B)** 1.5 Y_vs_3.5 Y; **(C)** 3.5 Y_vs_6 Y; **(D)** 4 M_vs_6 Y.

Procrustes analysis found that there were certain differences in rumen microbiota and metabolites of Tibetan sheep at different ages (Supplementary Figure S3). Correlation analysis revealed a strong correlation between differential metabolites in different age groups and genus-level microbiota. Screen |CC| > 0.8 and CCP < 0.05 data, the top 30 frequency differential metabolites/differential microbiota were made into a correlation chord diagram. In the 4 M_vs_1.5 Y (Figure 5A), a significant positive correlation was observed between *Lachnospiraceae_NK3A20_group* and metabolites (*p* < 0.05), while in the 1.5 Y_vs_3.5 Y (Figure 5B), a significant positive correlation was found between *Limosilactobacillus*, *Weissella*, and Cholesteryl glucoside (*p* < 0.05). In the 3.5 Y_vs_6 Y (Figure 5C), a significant positive correlation was observed between *Streptococcus* and Ginsenoside Rh5 (*p* < 0.05). In the 4 M_vs_6 Y (Figure 5D), a significant positive correlation was found between *Selenomonas* and L-Carnitine (*p* < 0.05).

### Interaction analysis between rumen microbial metabolome and host transcriptome (mRNA) in Tibetan sheep at different ages

**Figure 6:**
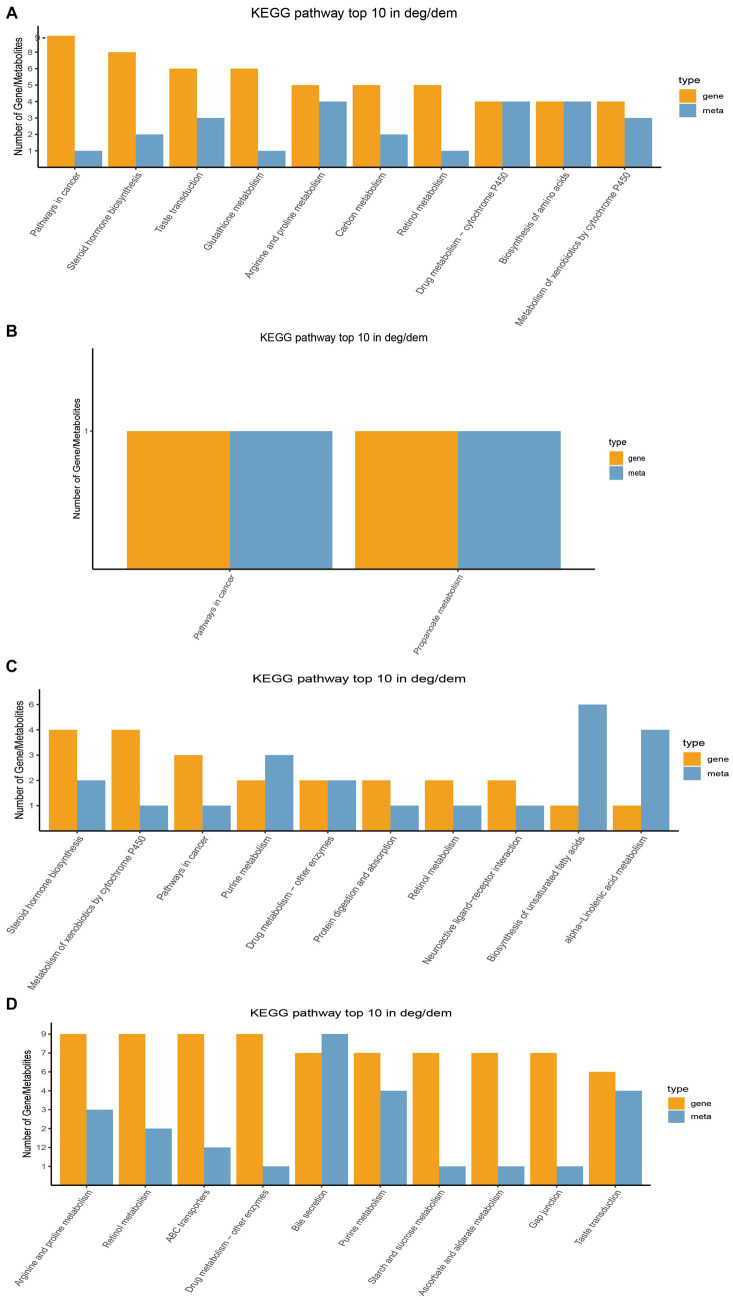
Top 10 co-enriched KEGG pathways for visual analysis. **(A)** 4 M_vs_1.5 Y; **(B)** 1.5 Y_vs_3.5 Y; **(C)** 3.5 Y_vs_6 Y; **(D)** 4 M_vs_6 Y.

Through transcriptome sequencing of rumen epithelial tissues, a total of 29,095 expressed genes were detected in the rumen epithelial tissues of Tibetan sheep at different ages, and 1,007 differentially expressed genes were screened out. WGCNA dimension reduction analysis divided genes and metabolites into different modules, and found that there was a certain correlation between these gene modules and metabolite modules (Supplementary Figure S4). Co-participating pathways were obtained by comparing pathways involved by genes in the transcriptome and pathways involved by metabolites in the metabolome (Supplementary Figure S5). We found 58 common pathways in 4 M_vs_1.5 Y, 2 common pathways in 1.5 Y_vs_3.5 Y, 26 common pathways in 3.5 Y_vs_6 Y, and 89 common pathways in 4 M_vs_6 Y. The top 10 KEGG pathways with the largest number of common participations of differential genes and differential metabolites identified in this study were further counted for visual analysis. In 4 M_vs_1.5 Y (Figure 6A), the differential genes and differential metabolites were enriched in arginine and proline metabolism (ko00330), biosynthesis of amino acids (ko01230) and metabolism of xenobiotics by cytochrome P450 (ko00980) pathway. In 1.5 Y_vs_3.5 Y (Figure 6B), it was enriched in pathways in cancer (ko05200) and propanoate metabolism (ko00640). In 1.5 Y_vs_3.5 Y (Figure 6C), it was enriched metabolism of xenobiotics by cytochrome P450 (ko00980), and purine metabolism (ko00230). In 4 M_vs_6 Y (Figure 6D), it was mainly enriched in arginine and proline metabolism (ko00330), starch and sucrose metabolism (ko00500), gap junction (ko04540), bile secretion (ko04976).

### Analysis of blood metabolic profile of Tibetan sheep at different ages

There were some differences in blood metabolites of Tibetan sheep at different ages (Supplementary Figure S6; Supplementary Data S2). Four hundred and two differential metabolites were found in 4 M_vs_1.5 Y (Supplementary Figure S7A), among which Aflatoxin ExB2, dIDP, PC (14:0/0:0) were down-regulated at 1.5 years old, and Litebamine was up-regulated. In 1.5 Y_vs_3.5 Y (Supplementary Figure S7B), 203 differential metabolites were found, among which Solanidine, Kanosamine 6-phosphate, and 2-cis-abscisate were down-regulated at 3.5 Y. In 3.5 Y_vs_6 Y (Supplementary Figure S7C), 209 differential metabolites were found, among which Biliverdin IX and Toxin T2 tetrol were up-regulated. Five hundred and forty differential metabolites were found in 4 M_vs_6 Y (Supplementary Figure S7D), among which Avermectin B1a aglycone was upregulated, N-Octanoyl-L-homoserine lactone, and Homocarnosine was downregulated.

**Figure 7:**
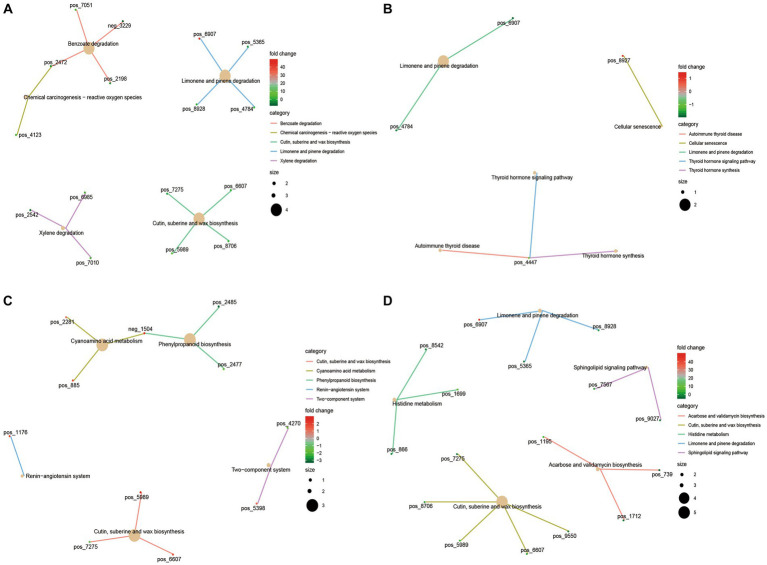
KEGG functional analysis of differential serum metabolites in Tibetan sheep at different ages. **(A)** 4 M_vs_1.5 Y; **(B)** 1.5 Y_vs_3.5 Y; **(C)** 3.5 Y_vs_6 Y; **(D)** 4 M_vs_6 Y.

The KEGG functional enrichment analysis of differential serum metabolites showed that benzoate degradation, chemical carcinogenesis − reactive oxygen species, cutin, suberine and wax biosynthesis were mainly enriched in 4 M_vs_1.5 Y (Figure 7A). In 1.5 Y_vs_3.5 Y (Figure 7B), it was mainly enriched in cellular senescence, Limonene and pinene degradation. In 3.5 Y_vs_6 Y (Figure 7C), it was mainly enriched in cyanoamino acid metabolism, cutin, suberine and wax biosynthesis. Histidine metabolism and sphingolipid signaling pathway were mainly enriched in 4 M_vs_6 Y (Figure 7D).

### Comparison and analysis of ruminal microbiota metabolic profile and blood metabolic profile

|log2FC| > 2 as the screening criteria, differential metabolites and serum metabolites were screened for comparative analysis. Twenty-one common differential metabolites were found in 4 M_vs_1.5 Y, no common metabolites were found between 1.5 Y_vs_3.5 Y, 4 common metabolites were found in 3.5 Y_vs_6 Y, and 49 common metabolites were found in 4 M_vs_6 Y (Supplementary Data S3). Further HMDB functional classification analysis of common differential metabolites (Figure 8) was carried out. Comparison between lambs, young, and old sheep found that the differential metabolites were mainly annotated in prenol lipids and organooxygen compounds. With only two metabolites annotation in glycerophospholipids. Compared to the lamb stage, Tyramine, Terpinen-4-ol, Leucomycin a5, Isoalantolactone, and Indole-3-carboxaldehyde were all downregulated in the rumen fluid and blood in both the youth and old stages. Salbostatin was downregulated in rumen fluid and upregulated in blood at young and old age. Anhwiedelphinine was upregulated in rumen fluid and blood at both adult and elderly stages.

**Figure 8:**
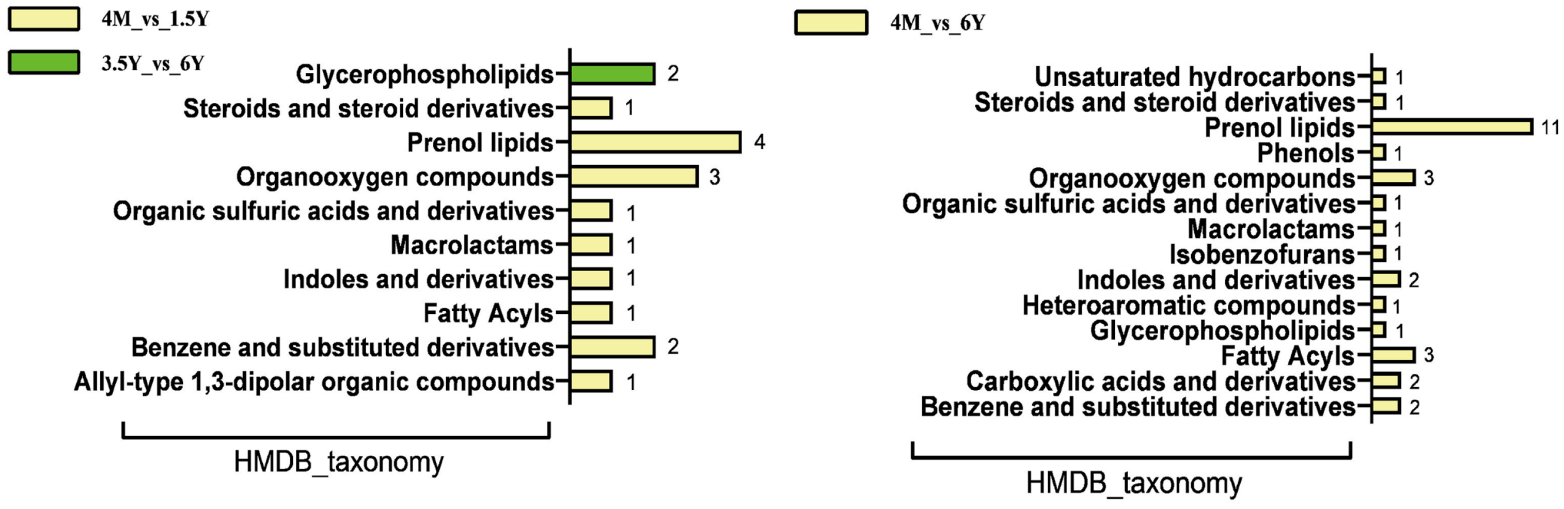
Common differential metabolites for HMDB functional classification analysis.

## Discussion

**Figure 9:**
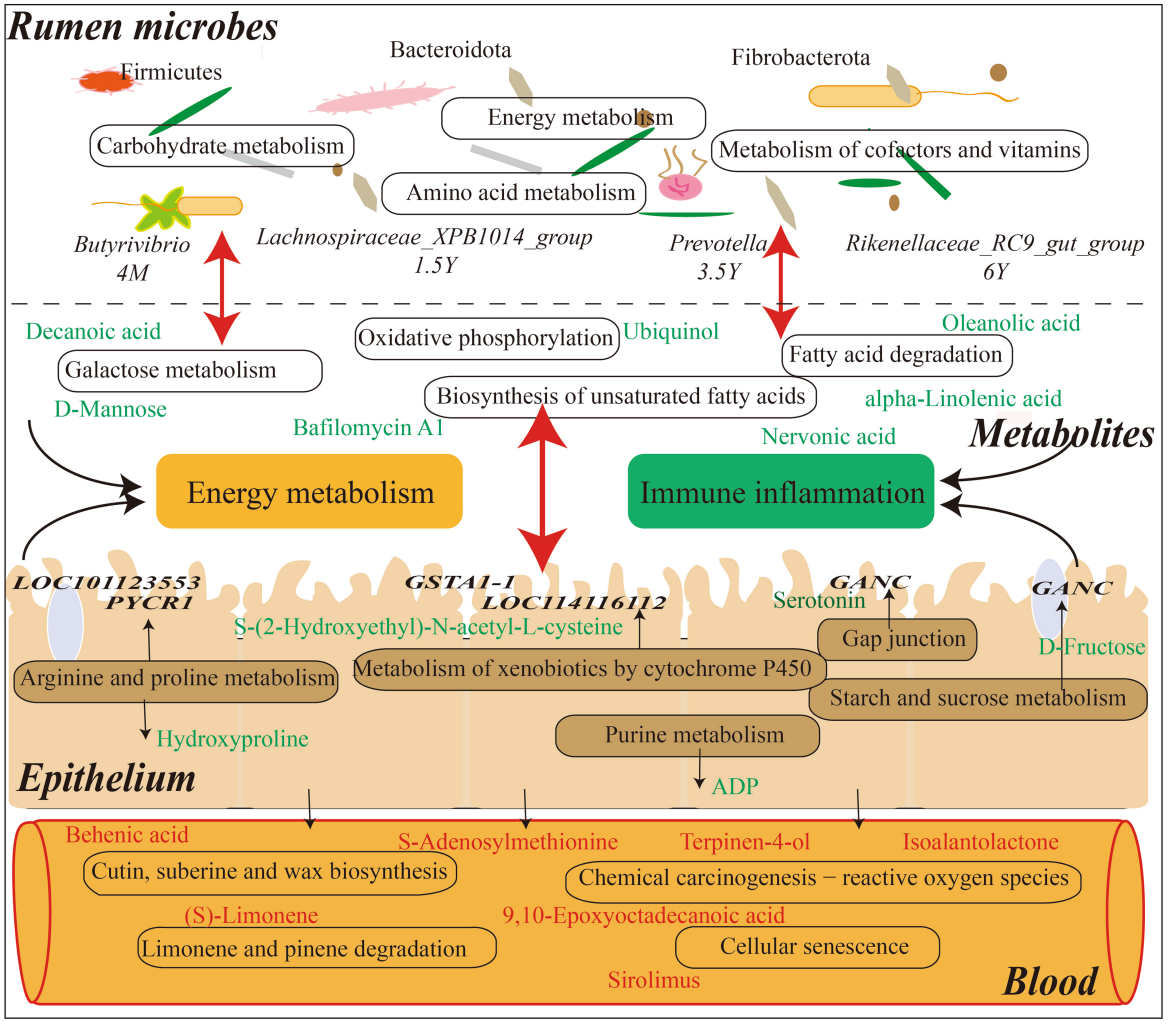
Interaction model diagram of rumen microorganisms and their metabolites with host mRNA and serum metabolites in Tibetan sheep at different ages.

Tibetan sheep is an important germplasm resource on the Qinghai-Tibet Plateau. The survival and production performance of Tibetan sheep in extreme environments are determined by the microbiota fermentation metabolism and host immune level in the rumen. The rumen microbiota composition and diversity of Tibetan sheep changed in the whole life stage from lamb to youth, adult and old age. The Shannon, ACE and Chao1 indexes of lamb stage were higher, indicating that the rumen microbial species abundance and diversity of Tibetan sheep at lamb stage were higher than those after adulthood. It was also found that *Firmicutes* (Kaakoush, 2015) related to digestion and absorption decreased with age and increased again in the old age, indicating that *Firmicutes* played an important role in nutrient absorption in the lamb stage and the old age stage. *Bacteroidota*, which is related to carbohydrate and protein degradation (Nuriel-Ohayon et al., 2016), has the highest abundance in the young and adult stages, and the lamb stage has a higher proportion of F/B, which helps the lamb to effectively absorb energy substances and maintain the metabolic balance of the body (Fernando et al., 2010; Murphy et al., 2010). Furthermore, the fiber-degrading bacterium *Fibrobacterota* (Ransom-Jones et al., 2012) increased in the old stage, and some digestive functions of Tibetan sheep are weakened in the old stage, so more *Fibrobacterota* may be needed to degrade the fiber material of pasture and provide energy material for the body. Microbial biomarker analysis found that *Butyrivibrio* decreased in the lamb stage, and the amount of *Butyrivibrio* was positively correlated with the amount of methanogen (Dan et al., 2016). More methane may be produced in the lamb stage, but the abundance of methane-degrading bacteria decreased with age. This is consistent with the low methane characteristics of plateau animals (Zhang et al., 2016). The biomarker bacterium *Lachnospiraceae_XPB1014_group* in the young stage is one of the producers of SCFAs and plays a role in host health (Vacca et al., 2020). *Prevotella*, the biomarker of adult stage, has the highest abundance in lambs and adult stages, which can produce more propionic acid (Strobel, 1992) to improve the performance of Tibetan sheep, which also confirms our previous research results (Sha et al., 2022). *Rikenellaceae_RC9_gut_group* in old age plays a role in the degradation of plant-derived polysaccharides (Seshadri et al., 2018), indicating that polysaccharides may be mainly degraded to provide energy substances for the old sheep. Further analysis of microbial KEGG function prediction found that carbohydrate metabolism was mainly in the lamb stage, amino acid metabolism and energy metabolism were increased in the young stage (Figure 9). The metabolism of cofactors and vitamins was enriched in the adult stage, indicating that the body needed more trace elements at this stage. In order to explore the specific microbial metabolic regulation pathways, we further measured the microbial metabolic profile.

The analysis of rumen microbial metabolic profile of Tibetan sheep at different ages found that decanoic acid (Kanabus et al., 2016; Dabke and Das, 2020) related to mitochondrial function and energy metabolism was higher in the lamb stage than in the young, so as to meet the energy required for rapid growth in the lamb stage. Moreover, 2-Succinylbenzoate increased during youth, serving as a menaquinone biosynthesis intermediate (Guest, 1977), and menaquinone plays a role in bacterial energy production and skeletal health (Johnston and Bulloch, 2020). In 1.5 Y_vs_3.5 Y, bafilomycin A1 content increased at the adult stage and was involved in the mitochondrial respiratory pathway under hypoxic conditions (Zhdanov et al., 2012), indicating that Tibetan sheep may have a stronger adaptability to the plateau hypoxic environment after adulthood. In 3.5 Y_vs_6 Y, oleanolic acid (Rodriguez-Rodriguez, 2015; Gutierrez et al., 2020), which is associated with inflammation, increases in the old stage and is used to prevent the occurrence of diseases. In 4 M_vs_6 Y, the compound megalomicin B (Volchegursky et al., 2000) with anti-parasitic, antiviral and antibacterial properties was found to be reduced in the old stage, indicating that the body’s disease resistance was weakened in the old stage. KEGG functional enrichment analysis of these differential metabolites showed that galactose metabolism and alpha−Linolenic acid metabolism were mainly enriched in 4 M_vs_1.5 Y (Figure 9). Among them, D-Mannose in galactose metabolism is upregulated at the lamb stage, which reduces the probability of inflammation (Torretta et al., 2020). And myo-Inositol (MacFarlane and Di Fiore, 2018), which is related to the respiratory system, is upregulated at the lamb stage. This may be a potentially important factor in the adaptation of Tibetan sheep to the plateau hypoxic environment in the lamb stage, which needs further verification. In 1.5 Y_vs_3.5 Y, ubiquinol, with higher content in the young stage, was enriched in the oxidative phosphorylation pathway, which is an important process in energy metabolism (Nolfi-Donegan et al., 2020). In 3.5 Y_vs_6 Y, the differential metabolites were mainly annotated in biosynthesis of unsaturated fatty acids, fatty acid degradation, among which nervonic acid was increased in the old age stage. It may be involved in the regulation of the body’s energy metabolism (Keppley et al., 2020) and maintain the energy demand in the old age. However, alpha-Linolenic acid (Yuan et al., 2022), a saturated fatty acid, is decreased in old age, indicating that fatty acid deposition capacity is reduced in old age and may be more used in energy metabolic processes. In 4 M_vs_6 Y, some down-regulated metabolites were found to be enriched in galactose metabolism, alpha−Linolenic acid metabolism and folate biosynthesis, which are some pathways related to energy metabolism. It indicates that the energy metabolism activity is more active in lamb stage. In addition, it was found that the cellular immune response marker neopterin (Michalak et al., 2017) was higher in the old stage than in the lamb stage, indicating that the immunity of the body was lower in the old stage than in the lamb stage. However, the changes of these metabolites are related to the structure of rumen microbiota. Further joint analysis found a significant positive correlation between *Lachnospiraceae_ NK3A20_group* and metabolites in 4 M_vs_1.5 Y. *Lachnospiraceae* can hydrolyze starch and other sugars to produce butyrate and other SCFAs (Biddle et al., 2013), and the abundance was highest in the lamb stage, high SCFA was provided for Tibetan sheep in the lamb stage, which was consistent with our previous research results (Sha et al., 2022). *Lachnospiraceae* not only provides energy substances, but also participates in the regulation of body health and disease (Vacca et al., 2020). Moreover, argatroban (McKeage and Plosker, 2001), which is related to thrombin, shows a significant positive correlation with the microbiota, contributing to the health of the lamb stage. In 1.5 Y_vs_3.5 Y, probiotics *Limosilactobacillus* and *Weissella* (Giraffa et al., 2010; Piccioni et al., 2021; Teixeira et al., 2021) were found to be significantly positively correlated with cholesteryl glucoside, which has a protective effect in gastric inflammation (Kunimoto et al., 2003), the content in youth is higher than that in adulthood. In 3.5 Y_vs_6 Y, *Streptococcus* (Mohammadi and Dhanashree, 2012) associated with lung disease was found to be significantly positively correlated with ginsenoside Rh5, which is an important bioactive compound and plays an important role in the occurrence of disease treatment (Dou et al., 2001; Li et al., 2022). It is increased in old Tibetan sheep and may play a role in disease prevention and treatment in old age. *Selenomonas* (Ricke et al., 1996), which is involved in carbohydrate catabolism and ATP generation pathways, was found to be significantly positively correlated with L-Carnitine in 4 M_vs_6 Y, and L-Carnitine is an endogenous molecule involved in fatty acid metabolism (Pekala et al., 2011), which is highest in lamb stage and may be involved in energy metabolism process.

Metabolites in the rumen are mainly transferred into the body through the rumen epithelium to play their functions. In this study, some common regulatory pathways were found through the joint analysis of the rumen microbial metabolome and the host transcriptome (Figure 9). In 4 M_vs_1.5 Y, it was found that the expression of *LOC101123553* and *PYCR1* in arginine and proline metabolism was down-regulated at young age, resulting in the increase of hydroxyproline. Studies have found that intestinal microbiota promote host resistance to low temperature stress by stimulating arginine and proline metabolic pathways (Raza et al., 2020). For Tibetan sheep, this pathway may improve adaptability in extreme plateau environment, and affect the downstream pentose and glucuronate interconversions pathway by regulating hydroxyproline to regulate the concentration and supply of intracellular sugars. And the differential genes enriched in metabolism of xenobiotics by cytochrome P450, such as *GSTA1-1* and *LOC114116112*, were highly expressed in the lamb stage. As a result, the content of pathway metabolite S-(2-Hydroxyethyl)-N-acetyl-L-cysteine is increased in the lamb stage, thus regulating the cell metabolism and homeostasis of the host (Zhao et al., 2021). In 1.5 Y_vs_3.5 Y, it is mainly enriched in propanoate metabolism, which may be involved in the regulation of intestinal epithelial development (Duan et al., 2023). In the 3.5 Y_vs_6 Y, differential genes such as *GSTA1-1* and *LOC114116112* in the metabolism of xenobiotics by cytochrome P450 pathway exhibited reduced expression levels in the old stage. This resulted in a decrease in the content of the metabolite S-(2-Hydroxyethyl)-N-acetyl-L-cysteine, indicating a decline in detoxification capacity and functional weakening in aspects such as cellular metabolism and internal balance in old Tibetan sheep (Zhao et al., 2021). As well as the content of ADP in purine metabolism, which is an energy substance produced by the decomposition of ATP (Tantama et al., 2013; Menegollo et al., 2019), increases in the old stage, indicating that energy demand is met through this pathway in the old stage. In 4 M_vs_6 Y, it was found that the high expression of *GANC* in starch and sucrose metabolism was involved in the regulation of D-Fructose content, which was higher in the lamb stage than in the old stage, thus providing energy for growth. In addition, the metabolite serotonin in the gap junction pathway increases in the old stage, and the receptor *HTR2B* is highly expressed. Studies have found that the enhancement of serotonin signaling in the gastrointestinal tract will lead to enhanced proliferation of intestinal epithelial cells and reduced intestinal inflammatory damage (Shah et al., 2021). In addition, the expression of *AOC1* and *ALDH9A1* in arginine and proline metabolism pathway is increased in the old stage, which is involved in butanoate metabolism pathway. *AOC1* gene is a marker of intestinal diseases (Liu et al., 2021), and its expression is higher in the old compared to the lamb stage.

Further analysis of the host serum metabolic profile showed that litebamine increased in young adults and was involved in platelet aggregation, ATP release and formation of thrombactin B2 (Teng et al., 1997; Huang et al., 2008), indicating enhanced antithrombotic activity in young adults. In 3.5 Y_vs_6 Y, an increase in biliverdin IX content in the old stage was found, which may be involved in anti-inflammatory response (Chen et al., 2012). And in 4 M_vs_6 Y, a decrease in homocarnosine content related to inflammatory treatment in the old stage was found (Huang et al., 2018). In 4 M_VS_1.5 Y, the differential serum metabolites were mainly enriched in cutin, suberine and wax biosynthesis, and the enriched behenic acid, a saturated fatty acid with elevated cholesterol, was down-regulated in the young stage (Cater and Denke, 2001). Secondly, it was enriched in chemical carcinogenesis − reactive oxygen species, among which S-adenosylmethionine was higher in lamb stage than in young stage. Studies have found that S-adenosylmethionine, as a methyl donor, mediates epigenetic effects to regulate the autophagy process (Ouyang et al., 2020), preventing the autophagy of Tibetan sheep in the plateau environment. In limonene and pinene degradation, related to oxidative stress and heart protection metabolites (Rhana et al., 2022) (S) - Limonene is higher were increased in lambs, and have anti-inflammatory, antioxidant (Pina et al., 2022) of the Carvone is also increased in lambs. In 1.5 Y_vs_3.5 Y, sirolimus up-regulated at the adult stage was enriched in cellular senescence and had antifungal, anti-proliferation and immunosuppressive activities (Sehgal, 2003), indicating that adult sheep had stronger adaptability to plateau. In 3.5 Y_vs_6 Y, the differential metabolites were mainly enriched in cyanoamino acid metabolism, cutin, suberine and wax biosynthesis. In cyanoamino acid metabolism, L-phenylalanine is up-regulated in the old stage, which is involved in the regulation of blood pressure (Wang et al., 2021), so as to better adapt to the plateau environment. In cutin, suberine and wax biosynthesis, behenic acid, a saturated fatty acid associated with elevated cholesterol (Cater and Denke, 2001), is downregulated in old age. 9, 10-epoxyoctadecanoic acid and 9, 10-dihydroxystearate were up-regulated, among which 9, 10-epoxyoctadecanoic acid is an oxide of oleic acid, which is the main fatty acid in mammals (Tsikas et al., 2003), this may provide energy for the old Tibetan sheep. Further comparative analysis of rumen metabolites and serum metabolites showed that the differential metabolites of prenol lipids, isoalantolactone and terpinen-4-ol, were higher in serum and rumen in lamb stage than in young and old stage. Studies have found that isoalantolactone has various biological activities such as anti-inflammatory and anti-oxidation (Xu et al., 2023), and terpinen-4-ol is also an antibacterial substance (Cordeiro et al., 2020), which plays a self-defense function through these metabolites in lamb Tibetan sheep. And tyramine (Andersen et al., 2019), which is associated with neuromodulation, cardiovascular and immune effects, are elevated in the lamb stage. In addition, salbostatin (Choi et al., 2008), produced by *Actinomyces* genus, was found to be down-regulated in rumen fluid and up-regulated in blood at young and old age. It was found that *Streptomyces* mainly produced some antibiotics (Worrall and Vijgenboom, 2010), which played an important role in body health, indicating that young and old Tibetan sheep produced some antibiotics through microbiotas to maintain their own health (Figure 9). These results reveal the different age paragraph the response characteristics of Tibetan sheep in plateau environment, provide a reference for different age groups of sheep breeding management.

## Data availability statement

The data presented in the study are deposited in the Sequence Read Archive (SRA) repository, accession number RJNA887585/PRJNA819418 (SRR18466104‐SRR18466108), and PRJNA1009954.

## Ethics statement

The animal studies were approved by Livestock Care Committee of Gansu Agricultural University (approval no. GAU-LC-2020-27). The studies were conducted in accordance with the local legislation and institutional requirements. Written informed consent was obtained from the owners for the participation of their animals in this study.

## Author contributions

YS: Conceptualization, Data curation, Formal analysis, Investigation, Methodology, Software, Writing – original draft. XL: Conceptualization, Funding acquisition, Investigation, Project administration, Supervision, Writing – review & editing. YH: Conceptualization, Supervision, Visualization, Writing – review & editing. SZ: Conceptualization, Writing – review & editing. JH: Project administration, Validation, Writing – review & editing. JW: Supervision, Writing – review & editing. WL: Investigation, Resources, Writing – review & editing. PS: Formal analysis, Investigation, Writing – review & editing. FW: Investigation, Methodology, Writing – review & editing. XC: Investigation, Resources, Writing – review & editing. WY: Investigation, Methodology, Writing – review & editing. ZX: Investigation, Resources, Writing – review & editing.

## Footnotes

^1^ www.biocloud.net

## References

[ref1] Andersen G. Marcinek P. Sulzinger N. Schieberle P. Krautwurst D. (2019). Food sources and biomolecular targets of tyramine. Nutr. Rev. 77, 107–115. doi: 10.1093/nutrit/nuy036, PMID: 30165672

[ref2] Aschenbach J. R. Penner G. B. Stumpff F. Gabel G. (2011). Ruminant nutrition symposium: role of fermentation acid absorption in the regulation of ruminal ph. J. Anim. Sci. 89, 1092–1107. doi: 10.2527/jas.2010-3301, PMID: 20952531

[ref3] Beharka A. A. Nagaraja T. G. Morrill J. L. Kennedy G. A. Klemm R. D. (1998). Effects of form of the diet on anatomical, microbial, and fermentative development of the rumen of neonatal calves. J. Dairy Sci. 81, 1946–1955. doi: 10.3168/jds.S0022-0302(98)75768-6, PMID: 9710764

[ref4] Bergman E. N. (1990). Energy contributions of volatile fatty acids from the gastrointestinal tract in various species. Physiol. Rev. 70, 567–590. doi: 10.1152/physrev.1990.70.2.567, PMID: 2181501

[ref5] Biddle A. Stewart L. Blanchard J. Leschine S. (2013). Untangling the genetic basis of fibrolytic specialization by lachnospiraceae and ruminococcaceae in diverse gut communities. Diversity 5, 627–640. doi: 10.3390/d5030627

[ref6] Cater N. B. Denke M. A. (2001). Behenic acid is a cholesterol-raising saturated fatty acid in humans. Am. J. Clin. Nutr. 73, 41–44. doi: 10.1093/ajcn/73.1.41, PMID: 11124748

[ref7] Chen H. Boutros P. C. (2011). Venndiagram: a package for the generation of highly-customizable Venn and Euler diagrams in R. BMC Bioinformatics. 12:35. doi: 10.1186/1471-2105-12-35, PMID: 21269502 PMC3041657

[ref8] Chen D. Brown J. D. Kawasaki Y. Bommer J. Takemoto J. Y. (2012). Scalable production of biliverdin ixalpha by *Escherichia coli*. BMC Biotechnol. 12:89. doi: 10.1186/1472-6750-12-89, PMID: 23176158 PMC3534565

[ref9] Choi W. S. Wu X. Choeng Y. H. Mahmud T. Jeong B. C. Lee S. H.. (2008). Genetic organization of the putative salbostatin biosynthetic gene cluster including the 2-epi-5-epi-valiolone synthase gene in *Streptomyces albus* ATCC 21838. Appl. Microbiol. Biotechnol. 80, 637–645. doi: 10.1007/s00253-008-1591-2, PMID: 18648803

[ref10] Cordeiro L. Figueiredo P. Souza H. Sousa A. Andrade-Junior F. Medeiros D.. (2020). Terpinen-4-ol as an antibacterial and antibiofilm agent against *Staphylococcus aureus*. Int. J. Mol. Sci. 21:4531. doi: 10.3390/ijms21124531, PMID: 32630600 PMC7350221

[ref11] Dabke P. Das A. M. (2020). Mechanism of action of ketogenic diet treatment: impact of decanoic acid and beta-hydroxybutyrate on sirtuins and energy metabolism in hippocampal murine neurons. Nutrients 12:2379. doi: 10.3390/nu12082379, PMID: 32784510 PMC7468807

[ref12] Dan X. Chen H. Chen F. He Y. Zhao C. Zhu D.. (2016). Analysis of the rumen bacteria and methanogenic archaea of yak (*Bos grunniens*) steers grazing on the Qinghai-Tibetan plateau. Livest. Sci. 188, 61–71. doi: 10.1016/j.livsci.2016.04.009

[ref13] Dou D. Q. Chen Y. J. Liang L. H. Pang F. G. Shimizu N. Takeda T. (2001). Six new dammarane-type triterpene saponins from the leaves of *Panax ginseng*. Chem. Pharm. Bull. 49, 442–446. doi: 10.1248/cpb.49.442 11310671

[ref14] Duan C. Wu J. Wang Z. Tan C. Hou L. Qian W.. (2023). Fucose promotes intestinal stem cell-mediated intestinal epithelial development through promoting akkermansia-related propanoate metabolism. Gut Microbes 15:2233149. doi: 10.1080/19490976.2023.2233149, PMID: 37424378 PMC10334863

[ref15] Dubois P. C. Trynka G. Franke L. Hunt K. A. Romanos J. Curtotti A.. (2010). Multiple common variants for celiac disease influencing immune gene expression. Nature Genet. 42, 295–302. doi: 10.1038/ng.543, PMID: 20190752 PMC2847618

[ref16] Dunn W. B. Broadhurst D. Begley P. Zelena E. Francis-McIntyre S. Anderson N.. (2011). Procedures for large-scale metabolic profiling of serum and plasma using gas chromatography and liquid chromatography coupled to mass spectrometry. Nat. Protoc. 6, 1060–1083. doi: 10.1038/nprot.2011.335, PMID: 21720319

[ref17] Edgar R. C. (2013). Uparse: highly accurate Otu sequences from microbial amplicon reads. Nat. Methods 10, 996–998. doi: 10.1038/nmeth.2604, PMID: 23955772

[ref18] Fernando S. C. Purvis H. N. Najar F. Z. Sukharnikov L. O. Krehbiel C. R. Nagaraja T. G.. (2010). Rumen microbial population dynamics during adaptation to a high-grain diet. Appl. Environ. Microbiol. 76, 7482–7490. doi: 10.1128/AEM.00388-10, PMID: 20851965 PMC2976194

[ref19] Giraffa G. Chanishvili N. Widyastuti Y. (2010). Importance of lactobacilli in food and feed biotechnology. Res. Microbiol. 161, 480–487. doi: 10.1016/j.resmic.2010.03.001, PMID: 20302928

[ref20] Gorka P. Kowalski Z. M. Pietrzak P. Kotunia A. Kiljanczyk R. Flaga J.. (2009). Effect of sodium butyrate supplementation in milk replacer and starter diet on rumen development in calves. J. Physiol. Pharmacol. 60, 47–53. 19996481

[ref21] Guest J. R. (1977). Menaquinone biosynthesis: mutants of *Escherichia coli* k-12 requiring 2-succinylbenzoate. J. Bacteriol. 130, 1038–1046. doi: 10.1128/jb.130.3.1038-1046.1977 324971 PMC235325

[ref22] Gutierrez B. Gallardo I. Ruiz L. Alvarez Y. Cachofeiro V. Margolles A.. (2020). Oleanolic acid ameliorates intestinal alterations associated with EAE. J. Neuroinflamm. 17:363. doi: 10.1186/s12974-020-02042-6, PMID: 33246492 PMC7697371

[ref23] Huang C. H. Huang W. J. Wang S. J. Wu P. H. Wu W. B. (2008). Litebamine, a phenanthrene alkaloid from the wood of *Litsea cubeba*, inhibits rat smooth muscle cell adhesion and migration on collagen. Eur. J. Pharmacol. 596, 25–31. doi: 10.1016/j.ejphar.2008.08.013, PMID: 18773889

[ref24] Huang J. Wang T. Yu D. Fang X. Fan H. Liu Q.. (2018). L-homocarnosine attenuates inflammation in cerebral ischemia-reperfusion injury through inhibition of nod-like receptor protein 3 inflammasome. Int. J. Biol. Macromol. 118, 357–364. doi: 10.1016/j.ijbiomac.2018.06.032, PMID: 29890246

[ref25] Jami E. Israel A. Kotser A. Mizrahi I. (2013). Exploring the bovine rumen bacterial community from birth to adulthood. ISME J. 7, 1069–1079. doi: 10.1038/ismej.2013.2, PMID: 23426008 PMC3660679

[ref26] Jing X. Wang W. Degen A. Guo Y. Kang J. Liu P.. (2020). Tibetan sheep have a high capacity to absorb and to regulate metabolism of SCFA in the rumen epithelium to adapt to low energy intake. Br. J. Nutr. 123, 721–736. doi: 10.1017/S0007114519003222, PMID: 31813386

[ref27] Johnston J. M. Bulloch E. M. (2020). Advances in menaquinone biosynthesis: sublocalisation and allosteric regulation. Curr. Opin. Struct. Biol. 65, 33–41. doi: 10.1016/j.sbi.2020.05.005, PMID: 32634692

[ref28] Kaakoush N. O. (2015). Insights into the role of erysipelotrichaceae in the human host. Front. Cell. Infect. Microbiol. 5:84. doi: 10.3389/fcimb.2015.00084, PMID: 26636046 PMC4653637

[ref29] Kanabus M. Fassone E. Hughes S. D. Bilooei S. F. Rutherford T. Donnell M. O.. (2016). The pleiotropic effects of decanoic acid treatment on mitochondrial function in fibroblasts from patients with complex i deficient leigh syndrome. J. Inherit. Metab. Dis. 39, 415–426. doi: 10.1007/s10545-016-9930-4, PMID: 27080638 PMC4851692

[ref30] Kanehisa M. Goto S. (2000). Kegg: Kyoto encyclopedia of genes and genomes. Nucleic Acids Res. 28, 27–30. doi: 10.1093/nar/28.1.27, PMID: 10592173 PMC102409

[ref31] Keppley L. Walker S. J. Gademsey A. N. Smith J. P. Keller S. R. Kester M.. (2020). Nervonic acid limits weight gain in a mouse model of diet-induced obesity. FASEB J. 34, 15314–15326. doi: 10.1096/fj.202000525R, PMID: 32959931 PMC8183615

[ref32] Kim M. H. Kang S. G. Park J. H. Yanagisawa M. Kim C. H. (2013). Short-chain fatty acids activate gpr41 and gpr43 on intestinal epithelial cells to promote inflammatory responses in mice. Gastroenterology 145, 396–406.e10. doi: 10.1053/j.gastro.2013.04.056, PMID: 23665276

[ref33] Kim D. Langmead B. Salzberg S. L. (2015). Hisat: a fast spliced aligner with low memory requirements. Nat. Methods 12, 357–360. doi: 10.1038/nmeth.3317, PMID: 25751142 PMC4655817

[ref34] Kristensen N. B. Harmon D. L. (2004). Effect of increasing ruminal butyrate absorption on splanchnic metabolism of volatile fatty acids absorbed from the washed reticulorumen of steers. J. Anim. Sci. 82, 3549–3559. doi: 10.2527/2004.82123549x, PMID: 15537776

[ref35] Kunimoto S. Murofushi W. Yamatsu I. Hasegawa Y. Sasaki N. Kobayashi S.. (2003). Cholesteryl glucoside-induced protection against gastric ulcer. Cell Struct. Funct. 28, 179–186. doi: 10.1247/csf.28.179, PMID: 12951438

[ref36] Li K. Li Z. Men L. Li W. Gong X. (2022). Potential of ginsenoside rh(2)and its derivatives as anti-cancer agents. Chin. J. Nat. Med. 20, 881–901. doi: 10.1016/S1875-5364(22)60193-6 36549803

[ref37] Li J. Ma W. Zeng P. Wang J. Geng B. Yang J.. (2015). Lnctar: a tool for predicting the RNA targets of long noncoding RNAs. Brief. Bioinform. 16, 806–812. doi: 10.1093/bib/bbu048, PMID: 25524864

[ref38] Li H. Yu Q. Li T. Shao L. Su M. Zhou H.. (2020). Rumen microbiome and metabolome of Tibetan sheep (Ovis Aries) reflect animal age and nutritional requirement. Front. Vet. Sci. 7:609. doi: 10.3389/fvets.2020.00609, PMID: 32984417 PMC7492597

[ref39] Liu F. Ou W. Tang W. Huang Z. Zhu Z. Ding W.. (2021). Increased aoc1 expression promotes cancer progression in colorectal cancer. Front. Oncol. 11:657210. doi: 10.3389/fonc.2021.657210, PMID: 34026633 PMC8131869

[ref40] Liu X. Sha Y. Lv W. Cao G. Guo X. Pu X.. (2022). Multi-omics reveals that the rumen transcriptome, microbiome, and its metabolome co-regulate cold season adaptability of Tibetan sheep. Front. Microbiol. 13:859601. doi: 10.3389/fmicb.2022.859601, PMID: 35495720 PMC9043902

[ref41] MacFarlane P. M. Di Fiore J. M. (2018). Myo-inositol effects on the developing respiratory neural control system. Adv.Exp.Med.Biol. 1071, 159–166. doi: 10.1007/978-3-319-91137-3_20, PMID: 30357747

[ref42] Martin R. Nauta A. J. Ben A. K. Knippels L. M. Knol J. Garssen J. (2010). Early life: gut microbiota and immune development in infancy. Benef. Microbes. 1, 367–382. doi: 10.3920/BM2010.0027 21831776

[ref43] McHardy I. H. Goudarzi M. Tong M. Ruegger P. M. Schwager E. Weger J. R.. (2013). Integrative analysis of the microbiome and metabolome of the human intestinal mucosal surface reveals exquisite inter-relationships. Microbiome. 1:17. doi: 10.1186/2049-2618-1-17, PMID: 24450808 PMC3971612

[ref44] McKeage K. Plosker G. L. (2001). Argatroban. Drugs 61, 515–522. doi: 10.2165/00003495-200161040-00005 11324681

[ref45] Menegollo M. Tessari I. Bubacco L. Szabadkai G. (2019). Determination of ATP, ADP, and AMP levels by reversed-phase high-performance liquid chromatography in cultured cells. Methods Mol. Biol. 1925, 223–232. doi: 10.1007/978-1-4939-9018-4_19, PMID: 30674030

[ref46] Michalak L. Bulska M. Strzabala K. Szczesniak P. (2017). Neopterin as a marker of cellular immunological response. Postep. Hig. Med. Dosw. 71, 01–736. doi: 10.5604/01.3001.0010.3851, PMID: 28894045

[ref47] Mohammadi J. S. Dhanashree B. (2012). *Streptococcus pseudopneumoniae*: an emerging respiratory tract pathogen. Indian J. Med. Res. 136, 877–880. PMID: 23287138 PMC3573612

[ref48] Murphy E. F. Cotter P. D. Healy S. Marques T. M. O'Sullivan O. Fouhy F.. (2010). Composition and energy harvesting capacity of the gut microbiota: relationship to diet, obesity and time in mouse models. Gut 59, 1635–1642. doi: 10.1136/gut.2010.215665, PMID: 20926643

[ref49] Nolfi-Donegan D. Braganza A. Shiva S. (2020). Mitochondrial electron transport chain: oxidative phosphorylation, oxidant production, and methods of measurement. Redox Biol. 37:101674. doi: 10.1016/j.redox.2020.101674, PMID: 32811789 PMC7767752

[ref50] Nuriel-Ohayon M. Neuman H. Koren O. (2016). Microbial changes during pregnancy, birth, and infancy. Front. Microbiol. 7:1031. doi: 10.3389/fmicb.2016.01031, PMID: 27471494 PMC4943946

[ref51] Ouyang Y. Wu Q. Li J. Sun S. Sun S. (2020). S-adenosylmethionine: a metabolite critical to the regulation of autophagy. Cell Prolif. 53:e12891. doi: 10.1111/cpr.12891, PMID: 33030764 PMC7653241

[ref52] Pekala J. Patkowska-Sokola B. Bodkowski R. Jamroz D. Nowakowski P. Lochynski S.. (2011). L-carnitine--metabolic functions and meaning in humans life. Curr. Drug Metab. 12, 667–678. doi: 10.2174/138920011796504536 21561431

[ref53] Piccioni A. Franza L. Vaccaro V. Saviano A. Zanza C. Candelli M.. (2021). Microbiota and probiotics: the role of limosilactobacillus reuteri in diverticulitis. Med. Lith. 57:802. doi: 10.3390/medicina57080802, PMID: 34441008 PMC8398895

[ref54] Pina L. Serafini M. R. Oliveira M. A. Sampaio L. A. Guimaraes J. O. Guimaraes A. G. (2022). Carvone and its pharmacological activities: a systematic review. Phytochemistry 196:113080. doi: 10.1016/j.phytochem.2021.113080, PMID: 34999510

[ref55] Ransom-Jones E. Jones D. L. McCarthy A. J. McDonald J. E. (2012). The fibrobacteres: an important phylum of cellulose-degrading bacteria. Microb. Ecol. 63, 267–281. doi: 10.1007/s00248-011-9998-1, PMID: 22213055

[ref56] Raza M. F. Wang Y. Cai Z. Bai S. Yao Z. Awan U. A.. (2020). Gut microbiota promotes host resistance to low-temperature stress by stimulating its arginine and proline metabolism pathway in adult *Bactrocera dorsalis*. PLoS Pathog. 16:e1008441. doi: 10.1371/journal.ppat.1008441, PMID: 32294136 PMC7185725

[ref57] Rey M. Enjalbert F. Combes S. Cauquil L. Bouchez O. Monteils V. (2014). Establishment of ruminal bacterial community in dairy calves from birth to weaning is sequential. J. Appl. Microbiol. 116, 245–257. doi: 10.1111/jam.12405, PMID: 24279326

[ref58] Rhana P. Barros G. M. Santos V. Costa A. D. Santos D. Fernandes-Braga W.. (2022). S-limonene protects the heart in an experimental model of myocardial infarction induced by isoproterenol: possible involvement of mitochondrial reactive oxygen species. Eur. J. Pharmacol. 930:175134. doi: 10.1016/j.ejphar.2022.175134, PMID: 35843301

[ref59] Ricke S. C. Martin S. A. Nisbet D. J. (1996). Ecology, metabolism, and genetics of ruminal selenomonads. Crit. Rev. Microbiol. 22, 27–65. doi: 10.3109/10408419609106455, PMID: 8729959

[ref60] Rodriguez-Rodriguez R. (2015). Oleanolic acid and related triterpenoids from olives on vascular function: molecular mechanisms and therapeutic perspectives. Curr. Med. Chem. 22, 1414–1425. doi: 10.2174/0929867322666141212122921, PMID: 25515513

[ref61] Segata N. Izard J. Waldron L. Gevers D. Miropolsky L. Garrett W. S.. (2011). Metagenomic biomarker discovery and explanation. Genome Biol. 12:R60. doi: 10.1186/gb-2011-12-6-r60, PMID: 21702898 PMC3218848

[ref62] Sehgal S. N. (2003). Sirolimus: its discovery, biological properties, and mechanism of action. Transplant. Proc. 35, S7–S14. doi: 10.1016/s0041-1345(03)00211-2 12742462

[ref63] Seshadri R. Leahy S. C. Attwood G. T. Teh K. H. Lambie S. C. Cookson A. L.. (2018). Cultivation and sequencing of rumen microbiome members from the hungate1000 collection. Nat. Biotechnol. 36, 359–367. doi: 10.1038/nbt.4110, PMID: 29553575 PMC6118326

[ref64] Sha Y. He Y. Liu X. Zhao S. Hu J. Wang J.. (2022). Rumen epithelial development- and metabolism-related genes regulate their micromorphology and VFAs mediating plateau adaptability at different ages in Tibetan sheep. Int. J. Mol. Sci. 23:16078. doi: 10.3390/ijms232416078, PMID: 36555715 PMC9786296

[ref65] Shabat S. K. Sasson G. Doron-Faigenboim A. Durman T. Yaacoby S. Berg M. M.. (2016). Specific microbiome-dependent mechanisms underlie the energy harvest efficiency of ruminants. ISME J. 10, 2958–2972. doi: 10.1038/ismej.2016.62, PMID: 27152936 PMC5148187

[ref66] Shah P. A. Park C. J. Shaughnessy M. P. Cowles R. A. (2021). Serotonin as a mitogen in the gastrointestinal tract: revisiting a familiar molecule in a new role. Cell. Mol. Gastroenterol. Hepatol. 12, 1093–1104. doi: 10.1016/j.jcmgh.2021.05.008, PMID: 34022423 PMC8350061

[ref67] Shen H. Xu Z. Shen Z. Lu Z. (2019). The regulation of ruminal short-chain fatty acids on the functions of rumen barriers. Front. Physiol. 10:1305. doi: 10.3389/fphys.2019.01305, PMID: 31749707 PMC6842973

[ref68] Sommer F. Backhed F. (2013). The gut microbiota--masters of host development and physiology. Nat. Rev. Microbiol. 11, 227–238. doi: 10.1038/nrmicro2974 23435359

[ref69] Strobel H. J. (1992). Vitamin b12-dependent propionate production by the ruminal bacterium *Prevotella ruminicola* 23. Appl. Environ. Microbiol. 58, 2331–2333. doi: 10.1128/aem.58.7.2331-2333.1992, PMID: 1637169 PMC195777

[ref70] Tantama M. Martinez-Francois J. R. Mongeon R. Yellen G. (2013). Imaging energy status in live cells with a fluorescent biosensor of the intracellular atp-to-adp ratio. Nat. Commun. 4:2550. doi: 10.1038/ncomms3550, PMID: 24096541 PMC3852917

[ref71] Teixeira C. G. Fusieger A. Miliao G. L. Martins E. Drider D. Nero L. A.. (2021). Weissella: an emerging bacterium with promising health benefits. Probiotics Antimicrob. Proteins. 13, 915–925. doi: 10.1007/s12602-021-09751-1 33565028

[ref72] Teng C. M. Hsueh C. M. Chang Y. L. Ko F. N. Lee S. S. Liu K. C. (1997). Antiplatelet effects of some aporphine and phenanthrene alkaloids in rabbits and man. J. Pharm. Pharmacol. 49, 706–711. doi: 10.1111/j.2042-7158.1997.tb06096.x, PMID: 9255715

[ref73] Tlaskalova-Hogenova H. Stepankova R. Kozakova H. Hudcovic T. Vannucci L. Tuckova L.. (2011). The role of gut microbiota (commensal bacteria) and the mucosal barrier in the pathogenesis of inflammatory and autoimmune diseases and cancer: contribution of germ-free and gnotobiotic animal models of human diseases. Cell. Mol. Immunol. 8, 110–120. doi: 10.1038/cmi.2010.67, PMID: 21278760 PMC4003137

[ref74] Torretta S. Scagliola A. Ricci L. Mainini F. Di Marco S. Cuccovillo I.. (2020). D-mannose suppresses macrophage il-1beta production. Nat. Commun. 11:6343. doi: 10.1038/s41467-020-20164-6, PMID: 33311467 PMC7733482

[ref75] Trapnell C. Williams B. A. Pertea G. Mortazavi A. Kwan G. van Baren M. J.. (2010). Transcript assembly and quantification by RNA-seq reveals unannotated transcripts and isoform switching during cell differentiation. Nat. Biotechnol. 28, 511–515. doi: 10.1038/nbt.1621, PMID: 20436464 PMC3146043

[ref76] Tremaroli V. Backhed F. (2012). Functional interactions between the gut microbiota and host metabolism. Nature 489, 242–249. doi: 10.1038/nature11552 22972297

[ref77] Tsikas D. Sawa M. Brunner G. Gutzki F. M. Meyer H. H. Frolich J. C. (2003). Gas chromatography-mass spectrometry of cis-9,10-epoxyoctadecanoic acid (cis-EODA). I. Direct evidence for cis-EODA formation from oleic acid oxidation by liver microsomes and isolated hepatocytes. J. Chromatogr. B 784, 351–365. doi: 10.1016/s1570-0232(02)00821-8 12505783

[ref78] Vacca M. Celano G. Calabrese F. M. Portincasa P. Gobbetti M. De Angelis M. (2020). The controversial role of human gut lachnospiraceae. Microorganisms. 8:573. doi: 10.3390/microorganisms8040573, PMID: 32326636 PMC7232163

[ref79] Volchegursky Y. Hu Z. Katz L. McDaniel R. (2000). Biosynthesis of the anti-parasitic agent megalomicin: transformation of erythromycin to megalomicin in *Saccharopolyspora erythraea*. Mol. Microbiol. 37, 752–762. doi: 10.1046/j.1365-2958.2000.02059.x, PMID: 10972798

[ref80] Wang Z. Cheng C. Yang X. Zhang C. (2021). L-phenylalanine attenuates high salt-induced hypertension in dahl ss rats through activation of gch1-bh4. PLoS One 16:e250126. doi: 10.1371/journal.pone.0250126, PMID: 33857222 PMC8049246

[ref81] Wang Y. Sheng H. F. He Y. Wu J. Y. Jiang Y. X. Tam N. F.. (2012). Comparison of the levels of bacterial diversity in freshwater, intertidal wetland, and marine sediments by using millions of Illumina tags. Appl. Environ. Microbiol. 78, 8264–8271. doi: 10.1128/AEM.01821-12, PMID: 23001654 PMC3497375

[ref82] Want E. J. Wilson I. D. Gika H. Theodoridis G. Plumb R. S. Shockcor J.. (2010). Global metabolic profiling procedures for urine using UPLC-MS. Nat. Protoc. 5, 1005–1018. doi: 10.1038/nprot.2010.50 20448546

[ref83] White J. R. Nagarajan N. Pop M. (2009). Statistical methods for detecting differentially abundant features in clinical metagenomic samples. PLoS Comput. Biol. 5:e1000352. doi: 10.1371/journal.pcbi.1000352, PMID: 19360128 PMC2661018

[ref84] Worrall J. A. Vijgenboom E. (2010). Copper mining in streptomyces: enzymes, natural products and development. Nat. Prod. Rep. 27, 742–756. doi: 10.1039/b804465c, PMID: 20372697

[ref85] Xu L. Sun Y. Cai Q. Wang M. Wang X. Wang S.. (2023). Research progress on pharmacological effects of isoalantolactone. J. Pharm. Pharmacol. 75, 585–592. doi: 10.1093/jpp/rgac103, PMID: 36940405

[ref86] Yin X. Ji S. Duan C. Tian P. Ju S. Yan H.. (2021). Age-related changes in the ruminal microbiota and their relationship with rumen fermentation in lambs. Front. Microbiol. 12:679135. doi: 10.3389/fmicb.2021.679135, PMID: 34616372 PMC8488279

[ref87] Yuan Q. Xie F. Huang W. Hu M. Yan Q. Chen Z.. (2022). The review of alpha-linolenic acid: sources, metabolism, and pharmacology. Phytother. Res. 36, 164–188. doi: 10.1002/ptr.7295, PMID: 34553434

[ref88] Zhang G. He P. Tan H. Budhu A. Gaedcke J. Ghadimi B. M.. (2013). Integration of metabolomics and transcriptomics revealed a fatty acid network exerting growth inhibitory effects in human pancreatic cancer. Clin. Cancer Res. 19, 4983–4993. doi: 10.1158/1078-0432.CCR-13-0209, PMID: 23918603 PMC3778077

[ref89] Zhang G. Wang Y. Luo H. Qiu W. Zhang H. Hu L.. (2019). The association between inflammaging and age-related changes in the ruminal and fecal microbiota among lactating Holstein cows. Front. Microbiol. 10:1803. doi: 10.3389/fmicb.2019.01803, PMID: 31447814 PMC6696898

[ref90] Zhang Z. Xu D. Wang L. Hao J. Wang J. Zhou X.. (2016). Convergent evolution of rumen microbiomes in high-altitude mammals. Curr. Biol. 26, 1873–1879. doi: 10.1016/j.cub.2016.05.012, PMID: 27321997

[ref91] Zhao M. Ma J. Li M. Zhang Y. Jiang B. Zhao X.. (2021). Cytochrome p450 enzymes and drug metabolism in humans. Int. J. Mol. Sci. 22:12808. doi: 10.3390/ijms222312808, PMID: 34884615 PMC8657965

[ref92] Zhdanov A. V. Dmitriev R. I. Papkovsky D. B. (2012). Bafilomycin a1 activates HIF-dependent signalling in human colon cancer cells via mitochondrial uncoupling. Biosci. Rep. 32, 587–595. doi: 10.1042/BSR20120085, PMID: 22943412 PMC3497721

