# Supplementary material for: Multi-omics revealed rumen microbiota metabolism and host immune regulation in Tibetan sheep of different ages
Source: Front Microbiol. 2024 Feb 13;15:1339889. doi: 10.3389/fmicb.2024.1339889 (PMC10896911; doi:10.3389/fmicb.2024.1339889)
Supplement: Supplementary file 4 [file Data_Sheet_1.DOCX]

*Frontiers in Microbiology*

**Multi-omics revealed rumen microbiota metabolism and host immune regulation in Tibetan sheep of different ages**

**Yuzhu Sha^1^, Xiu Liu^1,^*, Yanyu He^2^, Shengguo Zhao^1^, Jiang Hu^1^, Jiqing Wang^1^, Wenhao Li^3^, Pengyang Shao^1^, Fanxiong Wang^1^, Xiaowei Chen^1^, Wenxin Yang^1^, Zhuanhui Xie^1^**

^1^ College of Animal Science and Technology/Gansu Key Laboratory of Herbivorous Animal Biotechnology, Gansu Agricultural University, Lanzhou 730070, China

^2^ School of Fundamental Sciences, Massey University, Palmerston North 4410, New Zealand

^3^ Academy of Animal Science and Veterinary medicine, Qinghai University, Xining 810000, China

* Correspondence: [liuxiu@gsau.edu.cn](mailto:liuxiu@gsau.edu.cn), Tel.: +86‐931‐763‐1870

**Table. S1** Forage species and nutrient levels

| Nutrient composition (DM basis) | | Dominant forage species |
| --- | --- | --- |
| CP (%) | 10.06 | *Poa pratensis L* |
| EE (%) | 3.77 |  |
| Ash (%) | 4.55 | *Elymus nutans Griseb* |
| NDF (%) | 70.11 |  |
| DF (%) | 36.17 | *Agropyron cristatum (L.) Gaertn* |
| HCEL (%) | 33.94 |  |
| Ca (%) | 11.50 | *Stipa aliena Keng* |
| P (%) | 0.65 |  |
| Aboveground biomass（g/m^2^） | 343.52 | *Potentilla bifurca Linn.* |
| Grass height（cm） | 16.118 |  |


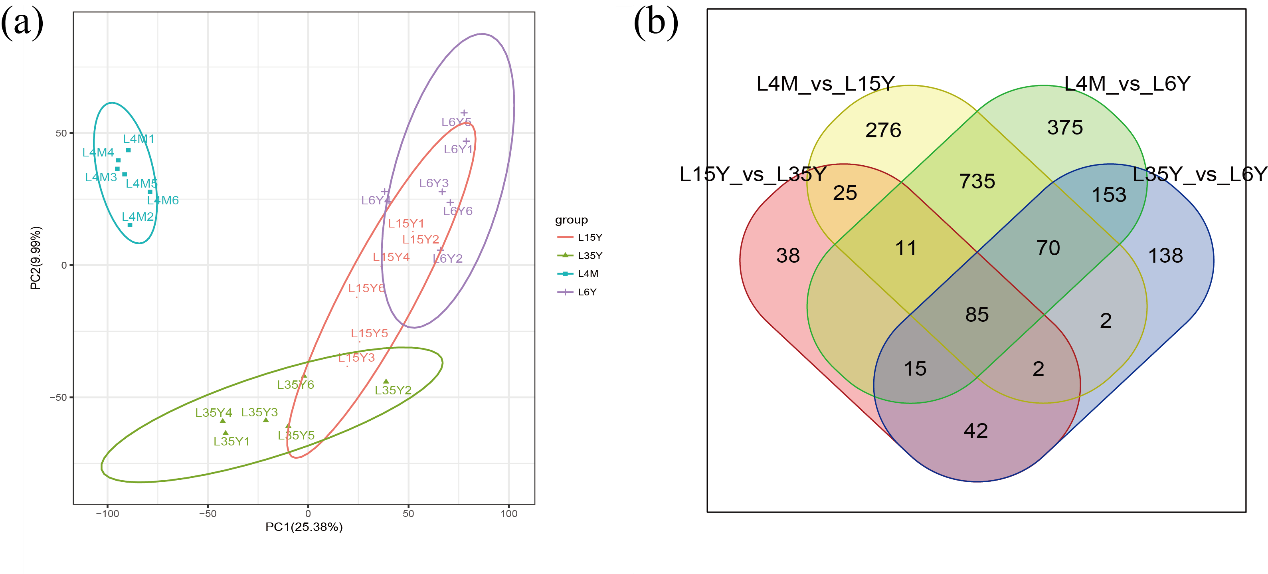


**Fig. S1** Rumen microbial metabolome analysis of Tibetan sheep at different ages. (a) PCA analysis; (b) Venn diagram of differential metabolites.


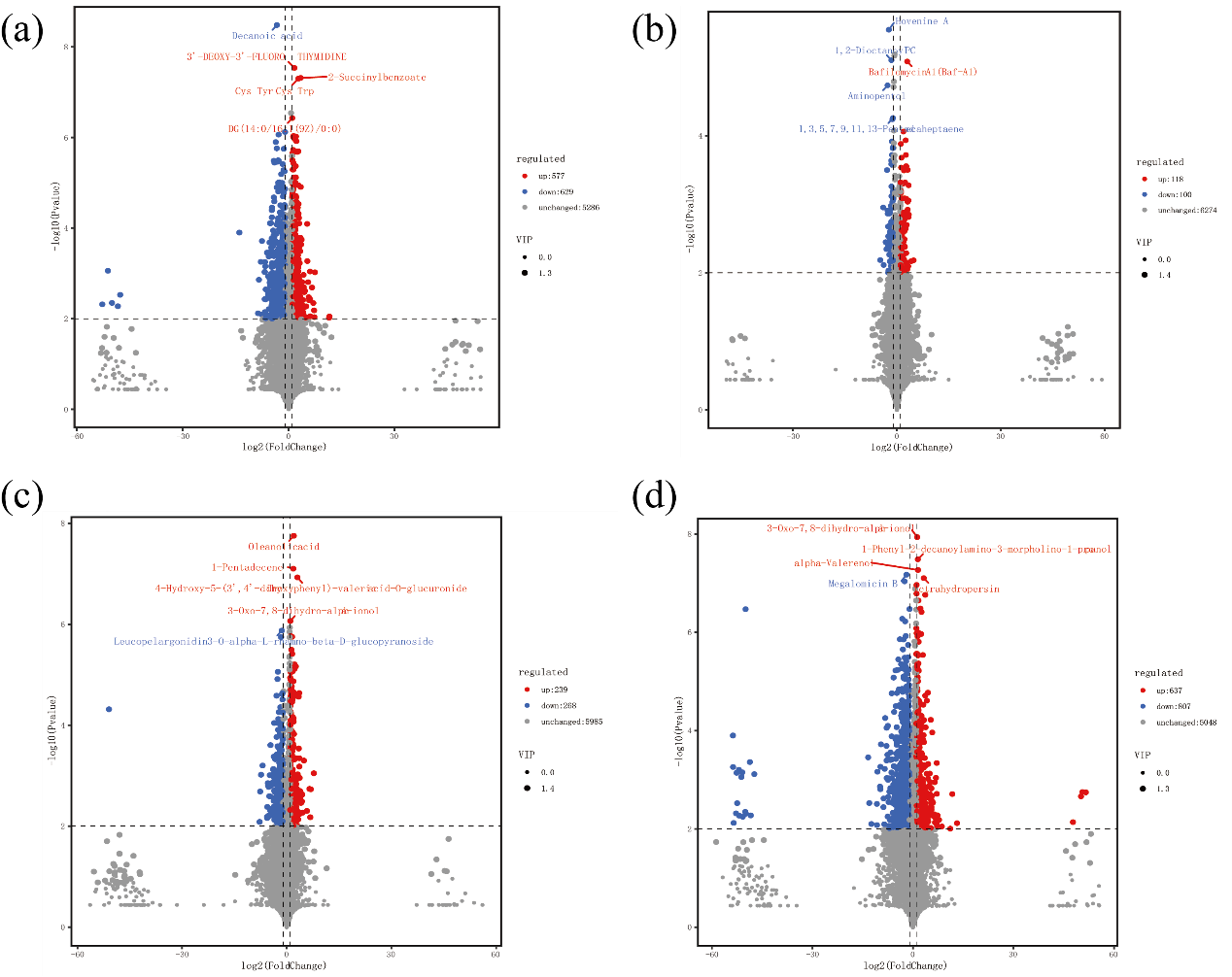


**Fig. S2** Volcanic map analysis of microbial metabolites in the rumen of Tibetan sheep at different ages. (a) 4M_vs_1.5Y; (b) 1.5Y_vs_3.5Y; (c) 3.5Y_vs_6Y; (d) 4M_vs_6Y.


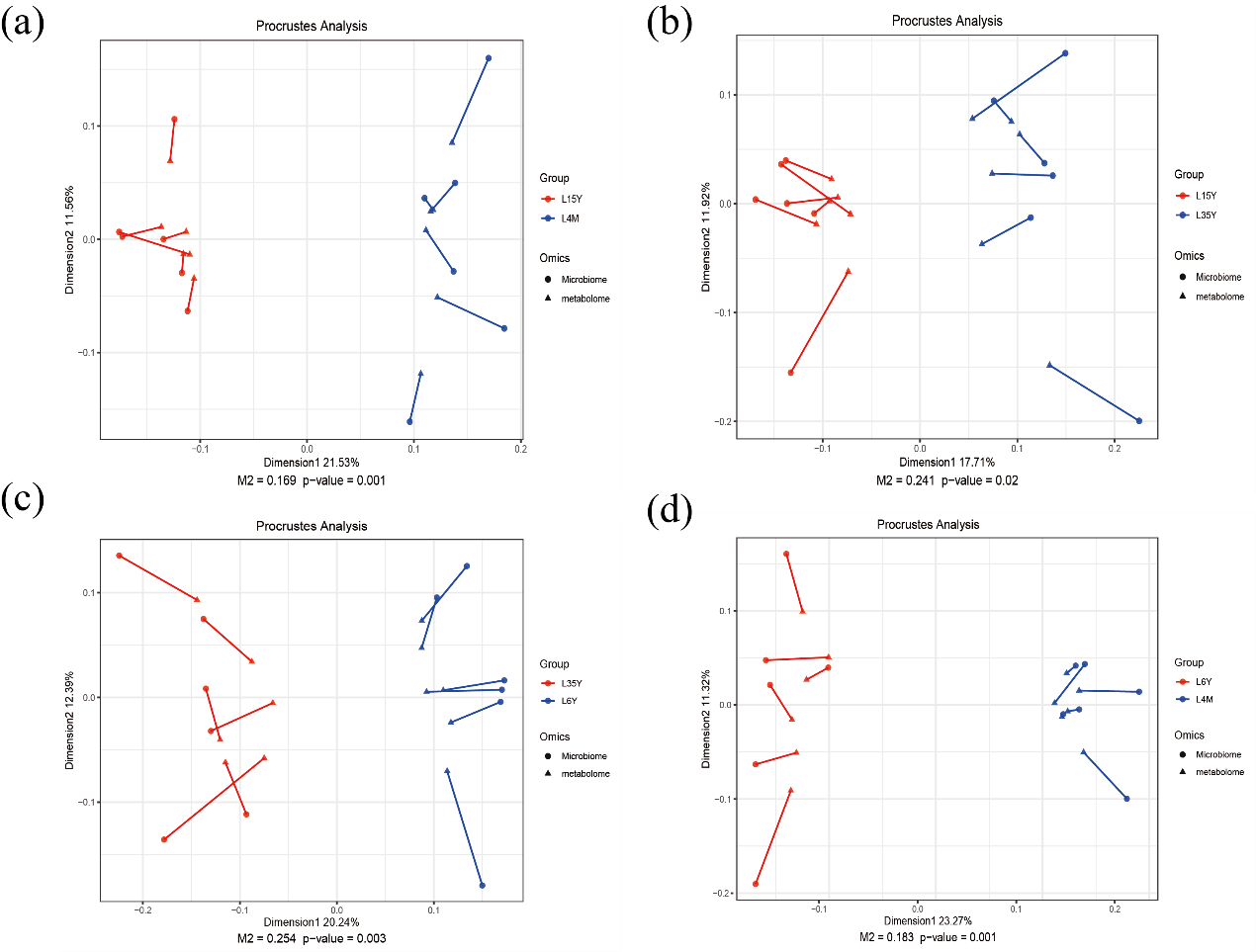


**Fig. S3** Ruminal microbe-metabolite Procrustes analysis. (a) 4M_vs_1.5Y; (b) 1.5Y_vs_3.5Y; (c) 3.5Y_vs_6Y; (d) 4M_vs_6Y.


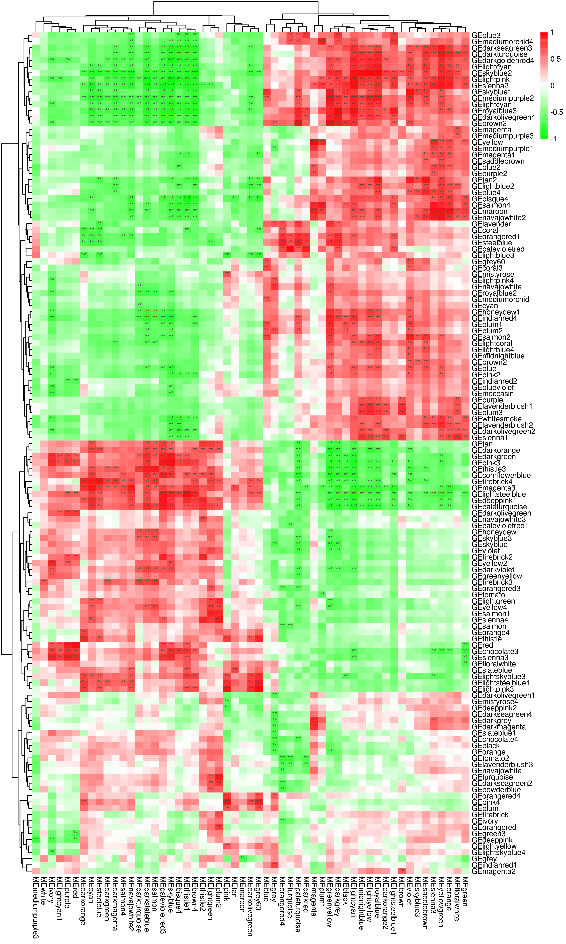

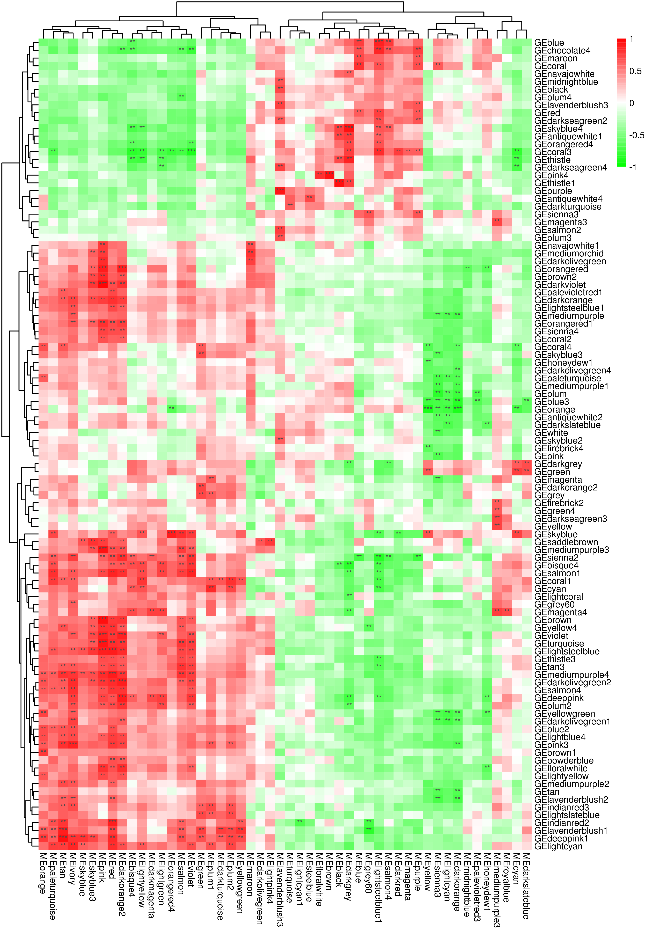


(a) (b)


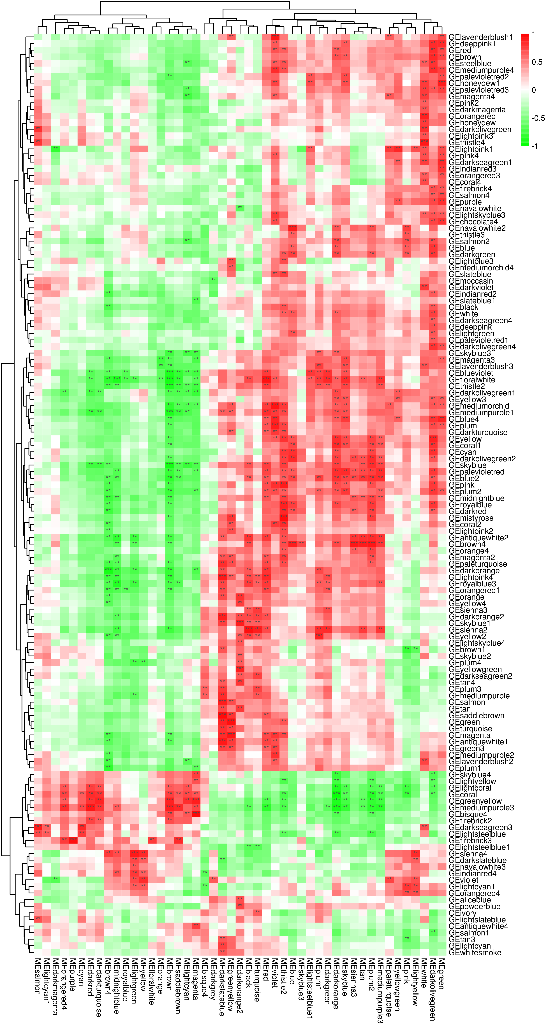

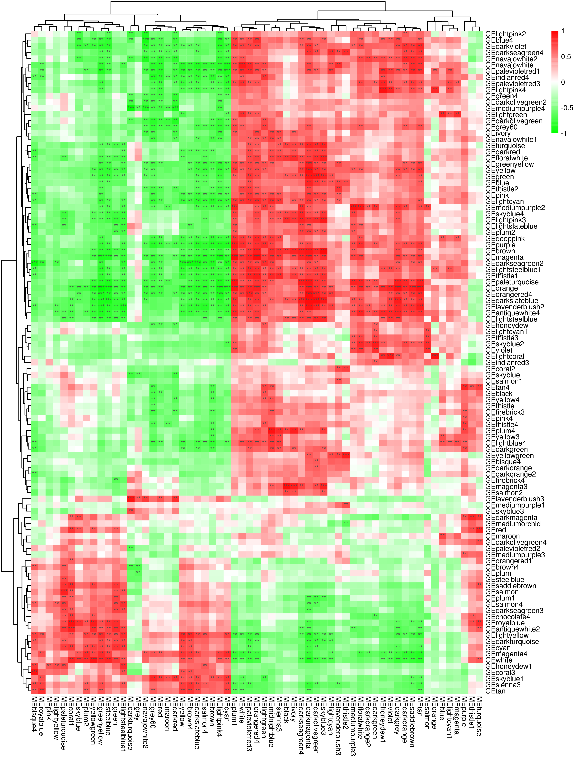


(c) (d)

**Fig. S4** WGCNA dimension reduction analysis. (a) 4M_vs_1.5Y; (b) 1.5Y_vs_3.5Y; (c) 3.5Y_vs_6Y; (d) 4M_vs_6Y.


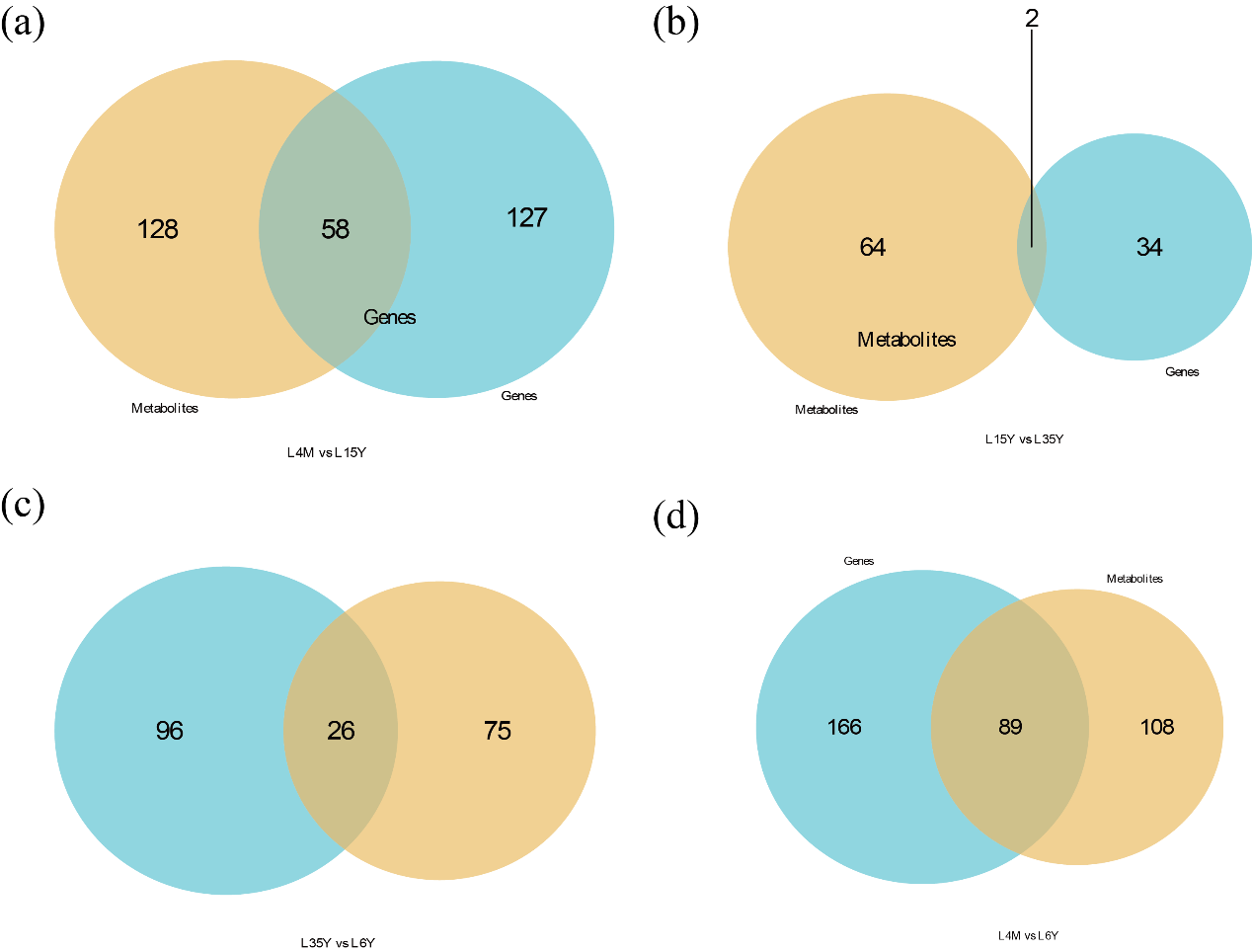


**Fig. S5** Statistics of co-enriched pathways for differential genes and differential metabolites. (a) 4M_vs_1.5Y; (b) 1.5Y_vs_3.5Y; (c) 3.5Y_vs_6Y; (d) 4M_vs_6Y.

**
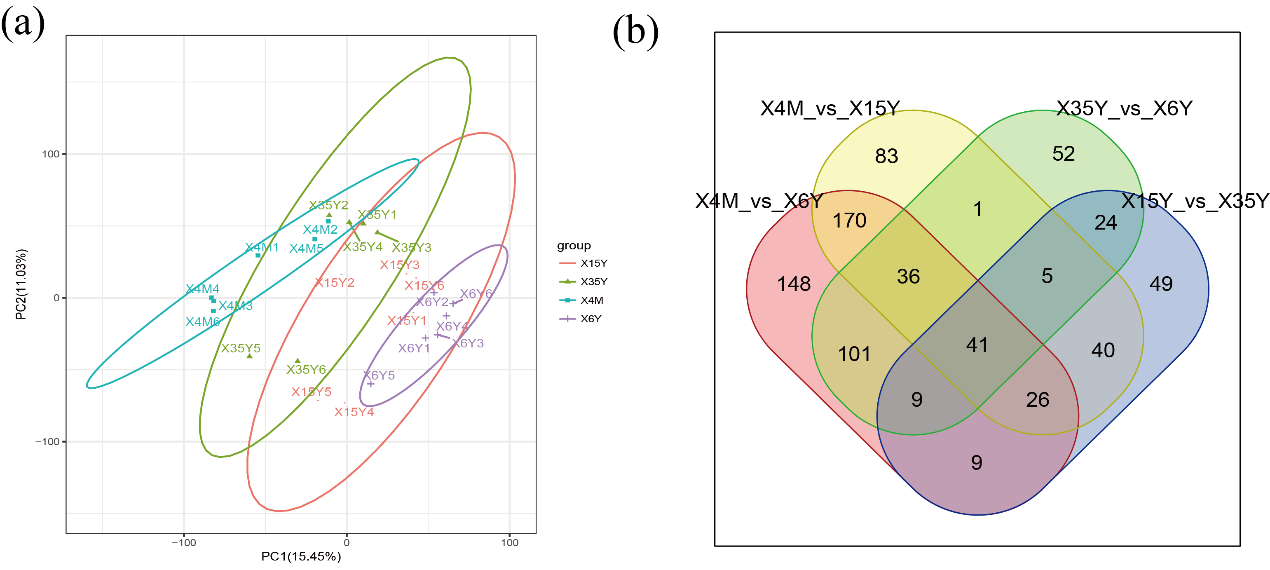
**

**Fig. S6** Analysis of serum metabolome of Tibetan sheep at different ages. (a) PCA analysis; (b) Venn diagram of differential metabolites.

**
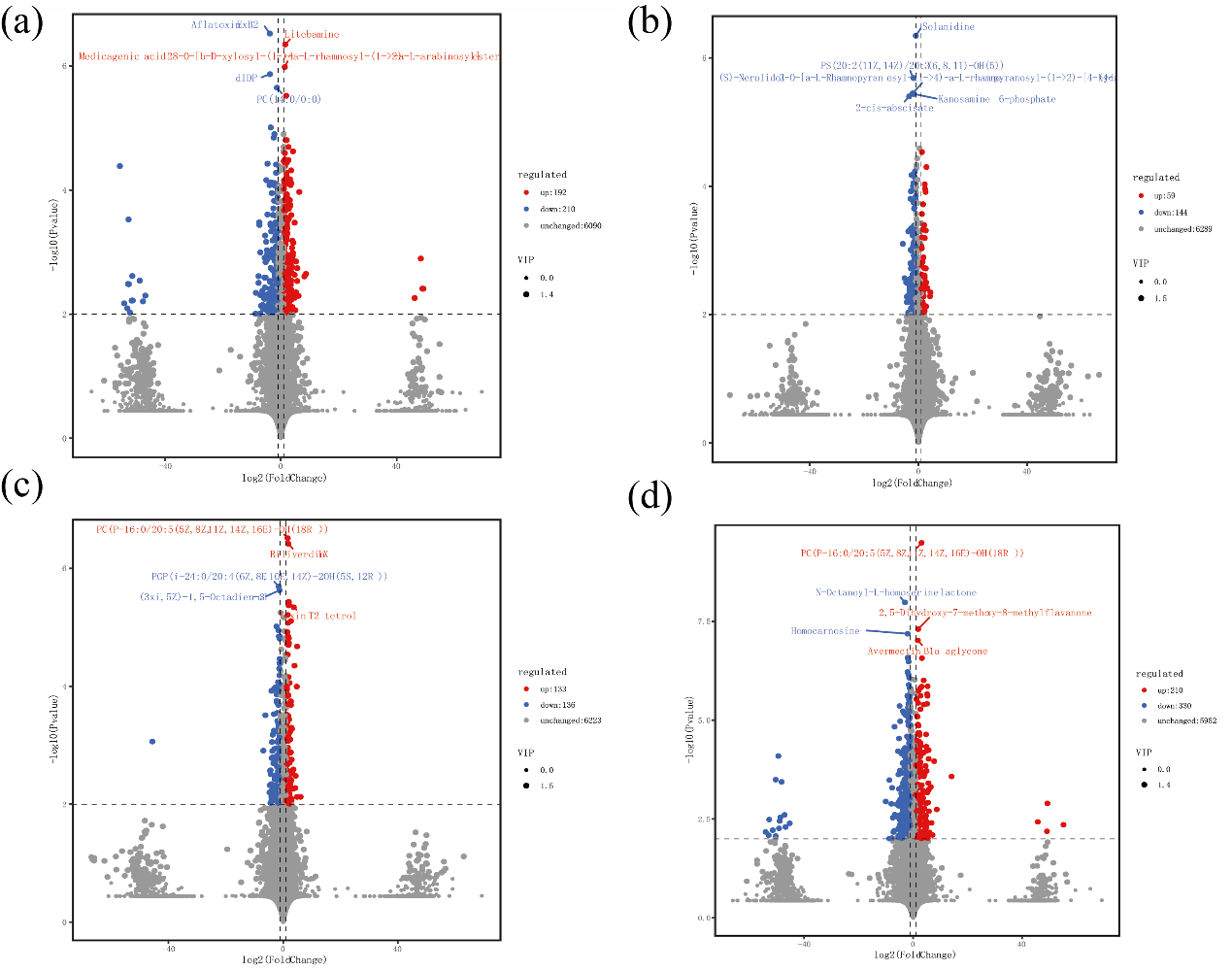
**

**Fig. S7** Volcano map analysis of serum metabolites in Tibetan sheep of different ages. (a) 4M_vs_1.5Y; (b) 1.5Y_vs_3.5Y; (c) 3.5Y_vs_6Y; (d) 4M_vs_6Y.
